# C-terminal Src Kinase Gates Homeostatic Synaptic Plasticity and Regulates Fasciclin II Expression at the Drosophila Neuromuscular Junction

**DOI:** 10.1371/journal.pgen.1005886

**Published:** 2016-02-22

**Authors:** Ashlyn M. Spring, Douglas J. Brusich, C. Andrew Frank

**Affiliations:** 1 Department of Anatomy and Cell Biology, University of Iowa Carver College of Medicine, Iowa City, Iowa, United States of America; 2 Interdisciplinary Graduate Program in Genetics, University of Iowa, Iowa City, Iowa, United States of America; 3 Interdisciplinary Programs in Genetics, Neuroscience, and MCB, University of Iowa, Iowa City, Iowa, United States of America; Stanford University School of Medicine, UNITED STATES

## Abstract

Forms of homeostatic plasticity stabilize neuronal outputs and promote physiologically favorable synapse function. A well-studied homeostatic system operates at the *Drosophila melanogaster* larval neuromuscular junction (NMJ). At the NMJ, impairment of postsynaptic glutamate receptor activity is offset by a compensatory increase in presynaptic neurotransmitter release. We aim to elucidate how this process operates on a molecular level and is preserved throughout development. In this study, we identified a tyrosine kinase-driven signaling system that sustains homeostatic control of NMJ function. We identified C-terminal Src Kinase (Csk) as a potential regulator of synaptic homeostasis through an RNAi- and electrophysiology-based genetic screen. We found that *Csk* loss-of-function mutations impaired the sustained expression of homeostatic plasticity at the NMJ, without drastically altering synapse growth or baseline neurotransmission. Muscle-specific overexpression of Src Family Kinase (SFK) substrates that are negatively regulated by Csk also impaired NMJ homeostasis. Surprisingly, we found that transgenic Csk-YFP can support homeostatic plasticity at the NMJ when expressed either in the muscle or in the nerve. However, only muscle-expressed Csk-YFP was able to localize to NMJ structures. By immunostaining, we found that *Csk* mutant NMJs had dysregulated expression of the Neural Cell Adhesion Molecule homolog Fasciclin II (FasII). By immunoblotting, we found that levels of a specific isoform of FasII were decreased in homeostatically challenged *GluRIIA* mutant animals–but markedly increased in *Csk* mutant animals. Additionally, we found that postsynaptic overexpression of FasII from its endogenous locus was sufficient to impair synaptic homeostasis, and genetically reducing FasII levels in *Csk* mutants fully restored synaptic homeostasis. Based on these data, we propose that Csk and its SFK substrates impinge upon homeostatic control of NMJ function by regulating downstream expression or localization of FasII.

## Author Summary

Homeostasis is a fundamental topic in biology. Individual cells and systems of cells constantly monitor their environments and adjust their outputs in order to maintain physiological properties within ranges that can support life. The nervous system is no exception. Synapses and circuits are endowed with a capacity to respond to environmental challenges in a homeostatic fashion. As a result, synaptic output stays within an appropriate physiological range. We know that homeostasis is a fundamental form of regulation in animal nervous systems, but we have very little information about how it works. In this study, we examine the fruit fly *Drosophila melanogaster* and its ability to maintain normal levels of synaptic output over long periods of developmental time. We identify new roles in this process for classical signaling molecules called C-terminal Src kinase, Src family kinases, as well as a neuronal cell adhesion molecule called Fasciclin II, which was previously shown to stabilize synaptic contacts between neurons and muscles. Our work contributes to a broader understanding of how neurons work to maintain stable outputs. Ultimately, this type of knowledge could have important implications for neurological disorders in which stability is lost, such as forms of epilepsy or ataxia.

## Introduction

Throughout a metazoan’s lifespan, its nervous system encounters numerous challenges to function. Responding to stress requires the flexibility afforded by forms of neuroplasticity. Yet even in plastic neurons, functional outputs of synapses must be kept within physiologically appropriate ranges. This type of control requires sensitive regulatory systems, including homeostatic forms of synaptic plasticity [1–7]. The precise molecular underpinnings of homeostatic neuroplasticity are elusive. Progress has been made at identifying individual molecules required for the homeostatic regulation of synapse function [1,6,7]. In addition, compelling links have been suggested between homeostatic synaptic plasticity and disease processes, such as epilepsy [8]. However, few factors have been organized into coherent signaling pathways.

The *Drosophila melanogaster* third instar larval neuromuscular junction (NMJ) is an ideal synapse for the study of homeostatic regulation [9]. At the NMJ, genetic and pharmacological manipulations can be employed to decrease the sensitivity of postsynaptic receptors to single vesicles of glutamate, thereby decreasing quantal size [9–11]. Decreased quantal size triggers retrograde (muscle-to-nerve), homeostatic signaling that drives increased evoked presynaptic neurotransmitter vesicle release–increased quantal content (QC). As a result, relatively normal levels of muscle excitation are maintained. We refer to this compensatory process as “synaptic homeostasis” or “NMJ homeostasis.”

Acute NMJ application of the postsynaptic glutamate receptor inhibitor philanthotoxin-433 (PhTox) induces a rapid decrease in quantal size and a sharp homeostatic increase of presynaptic neurotransmitter release on a short timescale (5–10 min) [10]. Since the retrograde and cellular signaling pathways that drive NMJ homeostatic plasticity are not well defined [9], PhTox has been exploited in screens to identify mutations that impair the rapid induction of synaptic homeostasis. What has emerged is an exquisite array of processes that dictates increases in QC through acute alterations in presynaptic Ca^2+^ influx, regulation of the size and replenishment of the readily releasable pool (RRP) of presynaptic vesicles, control of vesicle fusion events, and intrinsic neuronal excitability [10,12–23]. Changes in these Drosophila NMJ parameters resemble homeostatic processes that have been documented in mammalian synaptic preparations [24–29]. Therefore, diverse homeostatic processes likely share ancient, universally conserved mechanisms.

Less explored is the identity of molecules and signaling systems that sustain NMJ homeostasis throughout development. Studies indicate that some molecules required for the long-term maintenance of homeostatic plasticity at the NMJ are not required for the short-term induction of homeostatic signaling [9,30–33]. To address this issue in more depth, we combined RNA interference (RNAi)-based screening with genetics, electrophysiology, and synapse imaging [33]. Here we show that classical signaling molecules, including C-terminal Src Kinase (Csk) and Src family kinases (SFKs), and the trans-synaptic neural cell adhesion molecule Fasciclin II (FasII/NCAM) have roles in the long-term maintenance of synaptic homeostasis. Our data suggest Csk promotes homeostatic plasticity, at least in part, by repressing synaptic levels of FasII/NCAM. Our data could have implications for the strategies employed by synapses to facilitate long-term, trans-synaptic homeostatic signaling throughout development.

## Results

### Csk is required for the long-term maintenance of homeostatic plasticity at the NMJ

**Fig 1:**
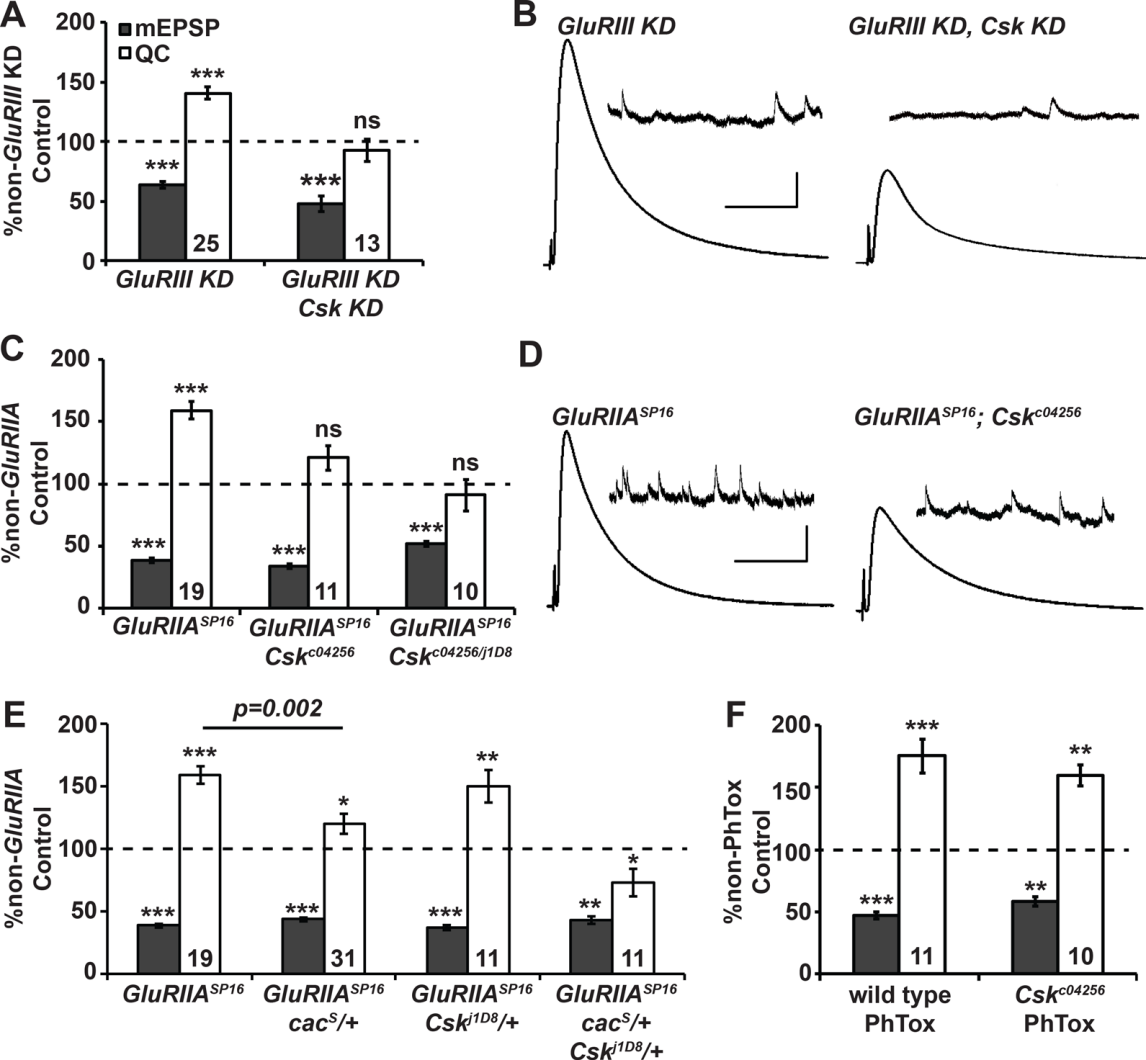
Csk is required for long-term homeostatic plasticity at the NMJ. **(A)** RNAi-mediated *Csk* knock down blocks synaptic homeostasis. Miniature excitatory postsynaptic potential amplitudes (mEPSP; gray) and quantal content (QC; white) normalized to genetic controls (dashed line) lacking a homeostatic challenge (non-*GluRIII* knockdown control). For both *GluRIII* and *Csk*, RNAi-mediated knock down is driven by the simultaneous presence of pre- and postsynaptic GAL4 drivers (see Methods and S1 Table for full genotypes). **(B)** Representative electrophysiological traces. Scale bar for EPSP (mEPSP): y = 5 mV (0.5 mV), x = 50 ms (1 s). **(C)** Normalized mEPSP and QC for *GluRIIA*^*SP16*^ mutant, *GluRIIA*^*SP16*^*; Csk*^*c04256*^ double mutant, and *GluRIIA*^*SP16*^*; Csk*^*c04256*^*/Csk*^*j1D8*^ double mutant NMJs. *GluRIIA; Csk* NMJs did not execute homeostatic increases in QC compared to baseline controls. **(D)** Representative electrophysiological traces. Scale bars as in B. **(E)** Normalized mEPSP and QC for *GluRIIA*^*SP16*^ mutant, *cac*^*S*^*/*+; *GluRIIA*^*SP16*^ mutant, *GluRIIA*^*SP16*^*; Csk*^*j1D8*^*/*+ double mutant, and *cac*^*S*^*/*+*; GluRIIA*^*SP16*^*; Csk*^*j1D8*^*/*+ triple mutant NMJs. **(F)** Normalized mEPSP and QC for *Csk*^*c02456*^ mutants treated with philanthotoxin-433 (PhTox). * *p* < 0.05, ** *p* < 0.01, *** *p* < 0.001, ns—not significant (*p* > 0.1) by Student’s T-test comparing homeostatically challenged mutants to their unchallenged (non-*GluRIII* KD, non-*GluRIIA*, or non-PhTox) genetic controls.

In an ongoing RNA interference (RNAi)-based screen to identify factors required to sustain homeostatic plasticity [33], we uncovered Csk as a potential regulator. For the screen, we impaired the expression of the *GluRIII* glutamate receptor subunit gene by RNAi, along with concurrent pre- and postsynaptic RNAi-mediated knockdown of screened target genes [33]. *GluRIII* knockdown on its own caused significantly diminished NMJ quantal size (mEPSP) (Fig 1A) [33]. Diminished quantal size was offset by a homeostatic increase in presynaptic neurotransmitter release (increased quantal content, QC) (Fig 1A; see S1 Table for raw electrophysiological data throughout manuscript) [33]. The response was similar to what has been shown previously for a *GluRIIA* glutamate receptor subunit deletion or mutation [11,34], acute philanthotoxin-433 (PhTox) application to the NMJ [10], and *GluRIII* genetic mutation [35]. By contrast, when the *GluRIII* and *Csk* genes were simultaneously knocked down by RNAi, larval progeny showed no significant increase in QC compared to *Csk[RNAi]* genetic controls (Fig 1A). This caused severely diminished evoked muscle excitation (Fig 1B). These initial findings suggested that Csk could be required for synaptic homeostasis.

We turned to loss-of-function mutations in *GluRIIA* and *Csk* to confirm the RNAi results. *GluRIIA*^*SP16*^ deletion NMJs have diminished quantal size, and the NMJ compensates for this defect with a homeostatic increase in quantal content (Fig 1C) [11]. We tested this canonical response for two independent, hypomorphic transposon insertions in the *Csk* locus, *Csk*^*c04256*^ and *Csk*^*j1D8*^ [36–39]. By electrophysiology, both *GluRIIA*; *Csk*^*c04256*^ double mutant NMJs and *GluRIIA; Csk*^*c04256*^*/Csk*^*j1D8*^ heteroallelic double mutant NMJs had expected decreases in quantal size compared to baseline *Csk* mutant genetic controls. However, neither had a homeostatic increase in QC (Fig 1C and 1D). This result was consistent with the RNAi screen findings.

For an additional test, we examined the effects of combining a heterozygous *Csk* mutation and a heterozygous *cacophony (cac)* mutation. The *cac* gene encodes the α1a subunit of the Drosophila Ca_V_2 calcium channel [40]. Cac is a key target of NMJ homeostatic regulation. Partial loss-of-function point mutations in *cac*, such as *cac*^*S*^, impair synaptic homeostasis [9,10,22,41]. Additionally, prior studies have documented strong heterozygous genetic interactions between *cac*^*S*^ and other mutations that disrupt synaptic homeostasis [19,30]. Consistent with these prior studies, the NMJs of *GluRIIA*; *cac*^*S*^*/+* heterozygotes showed a partial impairment of their homeostatic response (Fig 1E). Heterozygous *Csk*^*j1D8*^*/+* NMJs showed no significant defect in synaptic homeostasis (Fig 1E). However, when *cac*^*S*^*/+* and *Csk*^*j1D8*^*/+* mutant alleles were combined in a double heterozygous condition, the NMJs were completely unable to enhance quantal content in response to the *GluRIIA*^*SP16*^ mutation–in fact QC was slightly depressed compared to the non-*GluRIIA* mutant control (Fig 1E). These data indicate a strong genetic interaction exists between *cac* and *Csk* for proper homeostatic compensation.

### *Csk* mutations do not impair the rapid induction of synaptic homeostasis

Potentiation of Cac/Ca_V_2 function and enhancement of presynaptic calcium influx are critical for the rapid induction synaptic homeostasis [10,22]. Therefore, we needed to determine whether the Csk gene product also participates in this induction process–or if Csk primarily functions to maintain homeostatic signaling at the NMJ throughout development. Application of the glutamate receptor blocker PhTox to wild-type NMJs for 10 minutes is sufficient to impair glutamate receptor function, causing an acute diminishment in quantal size and a rapid, robust compensatory increase in quantal content (Fig 1F) [10]. Interestingly, rapid homeostatic plasticity remained fully intact when we applied PhTox to *Csk* mutant NMJs (Fig 1F). We conclude that Csk serves to maintain synaptic homeostasis throughout development, but appears to be dispensable for its acute induction.

### Csk mutant NMJs display normal growth and normal baseline neurotransmission

Our data suggested that Csk is required over developmental time for synaptic homeostasis to be executed properly. This could mean that Csk-mediated signaling pathways directly affect homeostatic regulation of neurotransmitter release throughout the course of development. However, it could also mean that depressed Csk levels disrupt homeostatic compensation and neurotransmission indirectly, by negatively impacting other biological processes such as NMJ growth.

**Fig 2:**
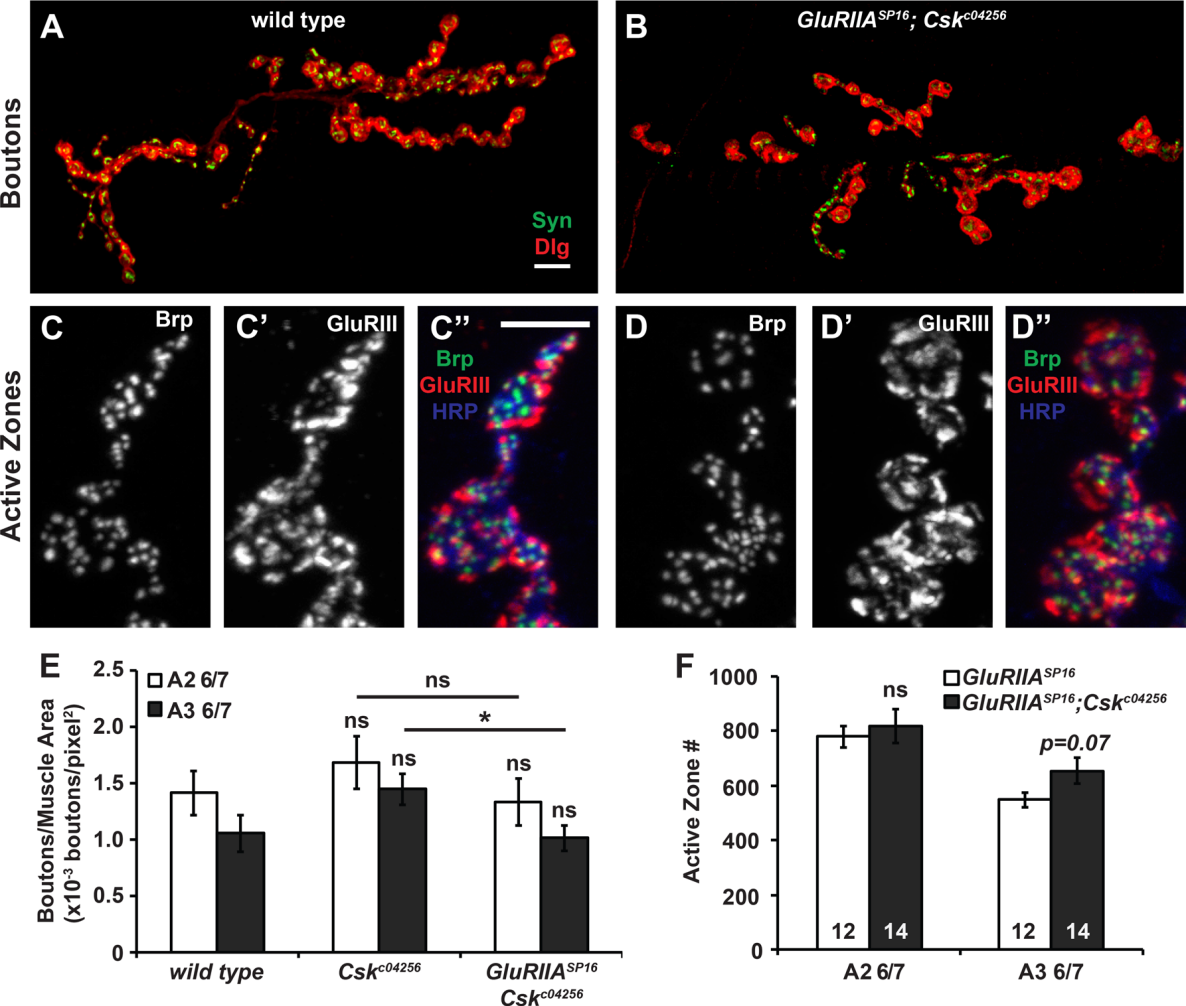
*Csk* mutant NMJs display normal growth. Bouton staining of **(A)** wild-type and **(B)** *GluRIIA*^*SP16*^*;Csk*^*c04256*^ NMJs. Boutons were stained presynaptically by anti-Synapsin (green) and postsynaptically by anti-Dlg (red). Scale bar = 10 μm. **(C-D”)** Active zone staining of **(C-C”)** *GluRIIA*^*SP16*^ and **(D-D”)** *GluRIIA*^*SP16*^*;Csk*^*c04256*^ mutant NMJs. The neuron is marked by HRP (blue), presynaptic active zones are marked by Brp (C, D, and green), and glutamate receptors are marked by GluRIII (C’, D’, and red). Scale bar = 5 μm. **(E)** Quantification of the bouton staining shown in A and B, muscle 6/7 synapse, segments A2 or A3 as indicated. Values shown are number of boutons per muscle area. See S1 Fig for non-normalized bouton number and muscle area. **(F)** Quantification of the active zone staining shown in C and D. Active zones were counted using Imaris 3D rendering software. * *p* < 0.05, ** *p* < 0.01, *** *p* < 0.001, ns—not significant (*p* > 0.1) by Student’s T-test.

To distinguish between these possibilities, we turned to immunofluorescence microscopy. First we quantified NMJ growth. We co-stained with antibodies that recognize the presynaptic vesicle protein Synapsin (anti-Syn) [42] and the postsynaptic PSD-95 scaffold Discs large (anti-Dlg) [43] and used the staining patterns to count NMJ boutons; then we normalized those counts per unit of muscle area. By these measures, NMJ growth was not significantly changed in *GluRIIA*; *Csk* mutant NMJs versus wild-type control NMJs (Fig 2A and 2B and 2E; see S1 Fig for all quantifications of bouton number, muscle size, and bouton count per unit muscle area). Therefore, severely impaired synapse growth is unlikely to be responsible for the defects in homeostatic plasticity of *GluRIIA; Csk* mutants. However, we note that bouton number per unit muscle area was decreased in segment A3, synapse 6/7 of *GluRIIA; Csk* double mutants versus *Csk* single mutants (Figs 2E and S1C). Therefore, defective NMJ development could play some role in impaired homeostatic compensation. To probe this issue further, we examined synaptic active zones.

We counted synaptic active zones after immunostaining with antibodies that recognize the presynaptic ELKS/CAST protein Bruchpilot (anti-Brp) [44] and the postsynaptic GluRIII glutamate receptor subunit [35]. For larval segment A2 synapse 6/7, we did not observe any significant changes in total active zone number at *GluRIIA*; *Csk* mutant NMJs compared to *GluRIIA* controls (Fig 2C–2C” and 2D–2D” and 2F). For larval segment A3 synapse 6/7, *GluRIIA*; *Csk* mutant NMJs displayed a slight (but statistically insignificant) increase in active zone number compared to *GluRIIA* controls (Fig 2F, *p* = 0.07, Student’s T-Test). Therefore, the defect in homeostatic plasticity cannot be explained a decrease in the number of synaptic active zone sites.

**Fig 3:**
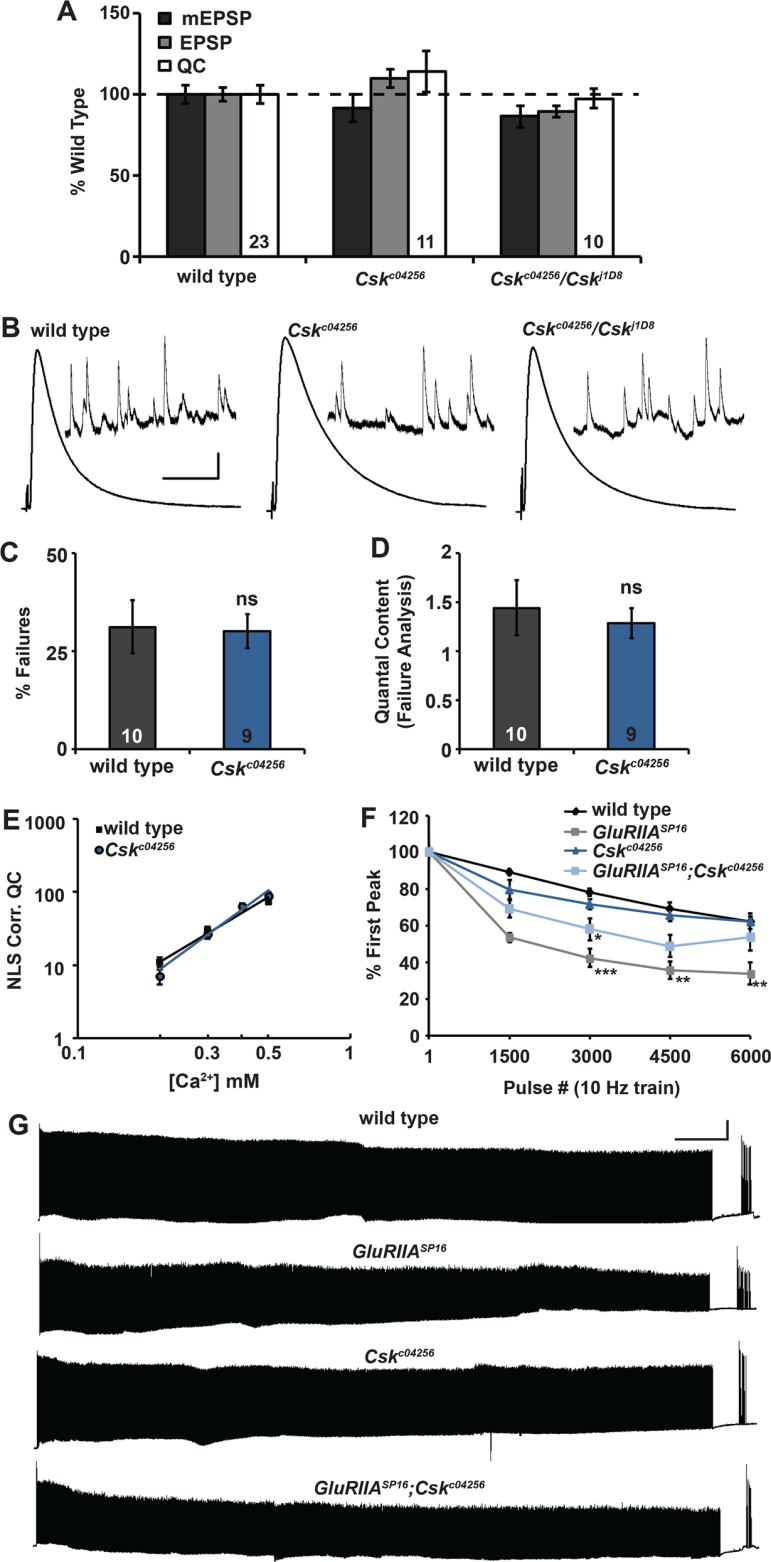
*Csk* mutant NMJs display normal baseline neurotransmission. **(A)** Values for mEPSP amplitude (black), EPSP amplitude (gray), and quantal content (QC; white) normalized to wild type (dashed line). No measures were significantly different from wild type. **(B)** Representative electrophysiological traces for the data shown in A. Scale bar for EPSP (mEPSP) traces: y = 5 mV (0.5 mV), x = 50 ms (1 s). **(C and D)** Failure analysis of wild type and *Csk* mutant NMJs. **(C)** % of total stimulus events that failed to produce an evoked response. **(D)** Quantal content calculated from failure analysis, QC = ln(# trials/# failures). **(E)** Calcium cooperativity curve from NMJs of wild type and *Csk* mutant animals. Quantal content corrected for non-linear summation (NLS) was determined at the extracellular calcium concentrations shown. **(F)** Average values for EPSP amplitudes as a percent of the initial EPSP amplitude at pulse 1, 1500, 3000, 4500, and 6000 of a 6000 pulse, 10 Hz train. **(G)** Representative electrophysiological traces for the data shown in F. Scale bar x = 1 min, y = 10 mV. For A-E, * *p* < 0.05, ** *p* < 0.01, *** *p* < 0.001, ns—not significant (*p* > 0.1) by Student’s T-test. For (F), * *p* < 0.05, ** *p* < 0.01, *** *p* < 0.001, ns—not significant (*p* > 0.1) by ANOVA (Tukey’s post-hoc) when comparing to wild type.

Next, we examined baseline neurotransmission at *Csk* mutant NMJs. Compared to wild-type NMJs, neither spontaneous miniature amplitudes (mEPSPs), nor evoked excitatory postsynaptic potentials (EPSPs), nor quantal content (QC) were different at homozygous *Csk*^*c04256*^ NMJs or heteroallelic *Csk*^*c04256*^*/Csk*^*j1D8*^ NMJs (S1 Table, Fig 3A and 3B). This result suggested that the electrophysiological defects we measured in *GluRIIA; Csk* double mutants (Fig 1C and 1D) were specifically due to problems in maintaining synaptic homeostasis.

We conducted a deeper characterization of baseline *Csk* electrophysiology. We previously demonstrated that *Csk/+* heterozygous mutations interact genetically with Ca_V_2 *cac*^*S*^*/+* heterozygous mutations to block synaptic homeostasis (Fig 1E). Therefore, we probed the sensitivity of *Csk* mutants to differential levels of extracellular Ca^2+^. First, we conducted failure analyses in extremely low calcium (0.14 mM) [10,11]. In low calcium, a significant proportion of evoked presynaptic events fail to release vesicles, resulting in stimulus artifacts, but no discernable EPSPs. Probability of vesicle release is low and quantal content is estimated as QC = ln([#trials/#failures]) [45]. By failure analysis, we found no effect on baseline probability of release for *Csk* mutant NMJs (Fig 3C and 3D). We also recorded from *Csk* mutant NMJs over a range of calcium concentrations. We also found no significant change in the calcium cooperativity of release (Fig 3E), suggesting that the calcium sensing machinery in the presynaptic cleft is functional in *Csk* mutants.

Finally, we probed the presynaptic vesicle pool and neurotransmitter release properties by challenging NMJs with a lengthy, high frequency stimulus paradigm (10 Hz x 6000 pulses = 10 minutes) in high extracellular calcium (2 mM). Under this condition, synapses with an elevated quantal content and release pool–as is the case with *GluRIIA* NMJs – significantly deplete their evoked responses over the course of such a train (Fig 3F and 3G) [10]. By contrast, *GluRIIA; Csk* double mutant NMJs did not deplete their responses to the same degree as *GluRIIA* NMJs (Fig 3F and 3G). By the end of the train, there was no excess depletion in *GluRIIA; Csk* double mutant NMJs compared to wild type or *Csk* mutant controls (Fig 3F and 3G). Importantly, after a short recovery time following the train (30 sec), all synapses analyzed displayed fully restored release (Fig 3G). Our data for *Csk* NMJs and for *GluRIIA; Csk* NMJs resemble prior examinations of NMJs that have an impaired long-term maintenance of homeostatic plasticity (e.g. [31]). Collectively, the immunostaining and electrophysiology data suggest a specific role in the long-term maintenance of synaptic homeostasis for Csk (Figs 1–3).

### Csk genetically opposes Src family kinases

The canonical function of Csk family members is to phosphorylate a C-terminal tyrosine residue in Src family kinases (SFKs) [46]. This phosphorylation event causes SFKs to assume an auto-inhibitory conformation, and it prevents them from phosphorylating downstream targets [46]. The negative Csk-SFK regulatory relationship is conserved in Drosophila. *In vivo* and *in vitro* assays have convincingly demonstrated that Drosophila Csk phosphorylates the Drosophila SFKs Src42A and Src64B at conserved tyrosines [37]. As a result, Drosophila Csk inhibits SFKs in processes such as cell growth and apoptosis [37,38]. It is plausible that Csk and SFKs employ the same type of regulatory relationship at NMJs. Indeed, by *in situ* hybridization (Berkeley Drosophila Genome Project data), enhancer trap analyses, and RNA sequencing analyses, these non-receptor tyrosine kinase genes have been shown to be expressed in many tissues in developing embryos and larvae–including larval central nervous systems and carcasses/musculature [47–53].

Based on those prior findings, the homeostatic defects caused by loss-of-function *Csk* mutations could be due to overactive SFK function. To test the possibility that overactive SFKs impair synaptic homeostasis, we acquired *UAS-SFK* transgenes: *UAS-Src64B*^*UY1332*^ [54], *UAS-Src42A*^*WT*^ [37], and *UAS-Src42A*^*YF*^ [37]. The *UAS-Src64B*^*UY1332*^ and *UAS-Src42A*^*WT*^ transgenes are wild type. The *UAS-Src42A*^*YF*^ transgene is constitutively active, as it lacks the inhibitory tyrosine residue 511 (Y511F) that is phosphorylated by Csk [37,55]. By combining *UAS-SFK* expression with a homozygous *GluRIIA*^*SP16*^ mutation, and tissue specific *GAL4* drivers, we examined how transgenic SFKs could impact NMJ homeostasis.

**Fig 4:**
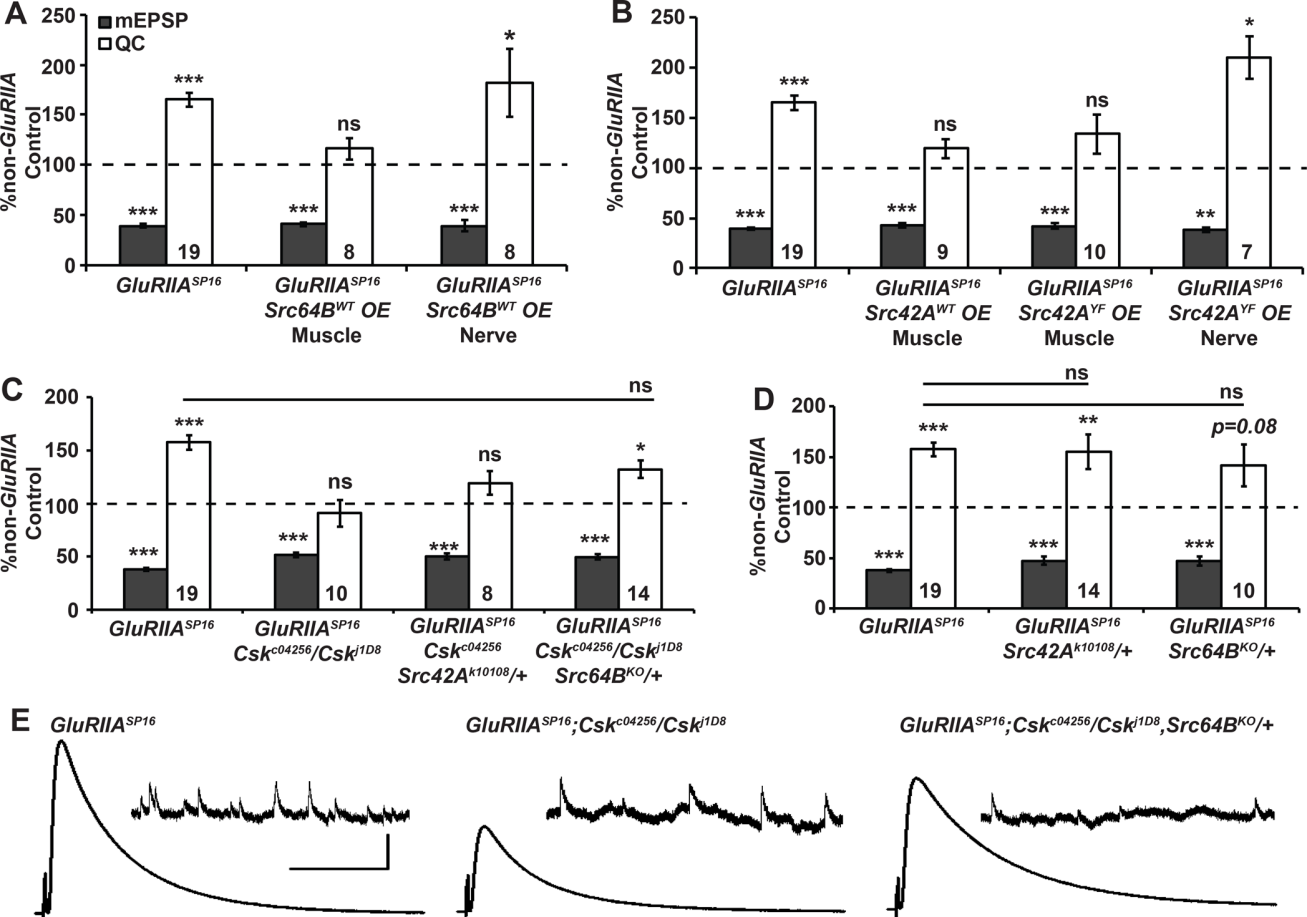
Csk genetically opposes Src family kinases in the context of synaptic homeostasis. **(A-D)** Values for mEPSP amplitude (gray) and quantal content (QC; white) normalized to genetic controls that lack a homeostatic challenge (non-*GluRIIA* Control, dashed line). **(A-B)** Muscle-specific SFK overexpression (OE) impairs synaptic homeostasis, while neuron-specific OE does not. **(C)** *Src64B/+* mutation partially suppresses the *GluRIIA; Csk* block of synaptic homeostasis. **(D)** *Src42A/+* and *Src64B/+* genetic conditions do not confer homeostatic defects on their own. **(E)** Representative electrophysiological traces for data shown in C. Scale bar for EPSP (mEPSP) traces: y = 5 mV (0.5 mV), x = 50 ms (1 s). * *p* < 0.05, ** *p* < 0.01 *** *p* < 0.001 ns—not significant (p > 0.1) by Student’s T-test of homeostatically challenged mutants directly to their unchallenged (non-*GluRIIA*) controls or by ANOVA (Tukey’s post-hoc) when comparing across multiple homeostatically-challenged genotypes in a dataset.

Expression of wild-type *UAS-Src64B*^*UY1332*^ in the muscle completely blocked homeostatic compensation (Fig 4A). By contrast, neuronal expression of wild-type *UAS-Src64B*^*UY1332*^ had no effect on the NMJ’s homeostatic capacity (Fig 4A). The effects of *Src42A* overexpression were similar to *Src64B*. Driving either the wild-type *UAS-Src42A*^*WT*^ transgene or the constitutively active *UAS-Src42A*^*YF*^ transgene in the muscle impaired synaptic homeostasis (Fig 4B). By contrast, neuronal expression of *UAS-Src42A*^*YF*^ had no inhibitory effect (Fig 4B). Our results are consistent with the possibility that a muscle-specific Csk/SFK regulatory interaction gates synaptic homeostasis. One qualification is that SFK overexpression had to be limited in order to circumvent embryonic and early larval lethality (see Materials and Methods and S1 Table for exact conditions for each SFK transgene). As a result, this experimental maneuver may have occluded potential Csk-SFK regulatory effects in neurons.

For a second approach, we checked if partial SFK loss could suppress the *GluRIIA; Csk* impairment of synaptic homeostasis. We acquired loss-of-function mutations in *Src42A* and *Src64B* [56–60]. To circumvent lethality, we analyzed heterozygous conditions. The heterozygous *Src42A*^*k10108*^*/+* condition increased average evoked amplitudes and QC in a *GluRIIA; Csk* double mutant background (S1 Table). However, the QC effect was not statistically significant (Fig 4C). By contrast, the heterozygous *Src64B*^*KO*^*/+* mutation did partially restore homeostatic capacity to *GluRIIA*; *Csk* double mutant NMJs (Fig 4C and 4E). Additionally, control experiments showed that *Src64B*^*KO*^*/+* caused no alterations in baseline neurotransmission on its own (S1 Table); nor did *GluRIIA; Src64B*^*KO*^*/+* NMJs significantly differ in homeostatic capacity compared to *GluRIIA* mutant NMJs (Fig 4D). Collectively, our SFK misexpression and mutant data support the idea that Csk and SFKs play antagonistic roles in the execution of homeostatic plasticity. This is consistent with findings in other organisms and Drosophila tissues.

### Either presynaptic or postsynaptic Csk is sufficient to support homeostatic plasticity

The SFK transgenic expression experiments suggested that a Csk-SFK regulatory process could direct the long-term maintenance of homeostatic compensation from the muscle. However, it is also possible that Csk serves regulatory functions in other tissues through other signaling modalities. Therefore, we performed transgenic *Csk* expression and rescue experiments. To do this, we generated *UAS-Csk-YFP* Drosophila stocks (see Materials and Methods), and we expressed Csk-YFP under *GAL4* control.

**Fig 5:**
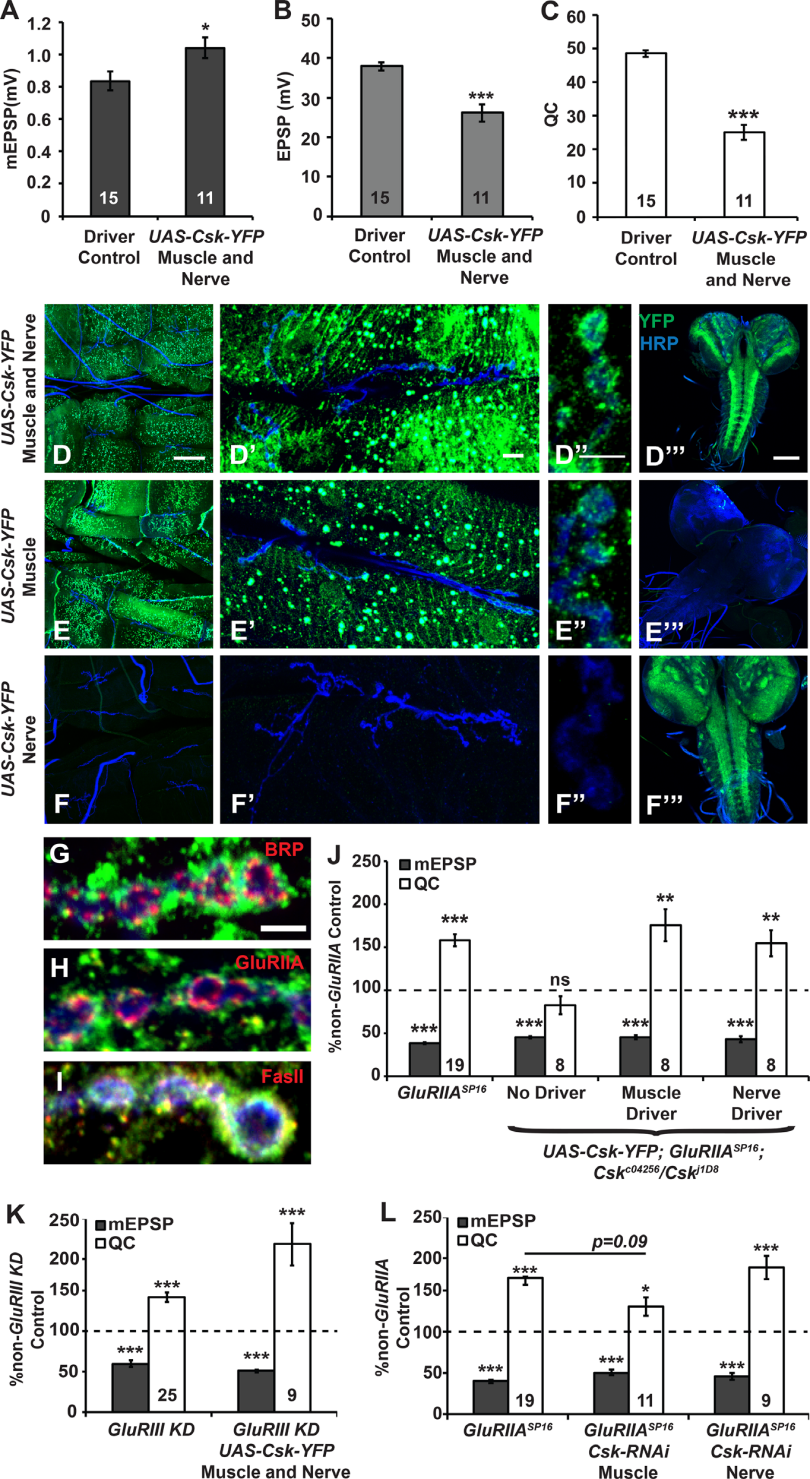
Transgenic Csk is sufficient for homeostatic compensation either presynaptically or postsynaptically. Average values for **(A)** mEPSP amplitude, **(B)** EPSP amplitude, and **(C)** quantal content (QC) for GAL4 driver control (concurrent *elaV(C155)-Gal4*, *Sca-Gal4*, *BG57-Gal4*) NMJs and those overexpressing YFP-tagged Csk on both sides of the synapse. **(D-F”‘)** Representative images of Csk-YFP localization at the NMJ and in the CNS, as detected by immunostaining when the construct is expressed on both sides of the synapse (D-D”), only in the muscle (E-E”), or only in neurons (F-F”‘). Neurons are marked by an anti-HRP antibody (blue) and Csk-YFP is shown in green. **(D, E, and F)** Images showing abdominal segments that include muscles 6/7 and images that show boutons at the NMJ. **(D’, E’, and F’)** Images showing a single muscle 6/7 synapse. **(D”, E”, and F”)** Images showing boutons from a muscle 6/7 synapse at high magnification. **(D”‘, E”‘, and F”‘)** Images showing the larval central nervous system. **(G-I)** Bouton localization of Csk-YFP (green) relative to Bruchpilot (Brp, G), the GluRIIA subunit (H), or Fasciclin II (FasII, I) at muscle 6/7 synapses at which Csk-YFP is expressed only in the muscle. **(J-L)** Average values for mEPSP amplitude (gray) and quantal content (QC; white) normalized to genetic controls (dashed line) that lack a homeostatic challenge (non-*GluRIIA* Control). * *p* < 0.05, ** *p* < 0.01, *** *p* < 0.001, ns—not significant (p > 0.1) by Student’s T-test of homeostatically challenged mutants to their unchallenged (non-*GluRIIA*) controls and by ANOVA (Tukey’s post-hoc) when comparing across multiple homeostatically-challenged genotypes in a dataset.

In a wild-type genetic background, concurrent muscle and nerve expression of Csk-YFP caused slight electrophysiological abnormalities, including a slight increase in quantal size (Fig 5A), but decreases in evoked postsynaptic excitation (Fig 5B) and quantal content (Fig 5C). We imaged concurrent muscle- and nerve-expressed Csk-YFP in unfixed, filleted larvae by fluorescence microscopy. We also used an anti-GFP antibody to stain fixed, filled larvae. In both cases, we observed a strong, punctate cytoplasmic Csk-YFP signal in the muscle (Fig 5D and 5D’). By immunostaining we could detect low levels of Csk-YFP clustered at the NMJ (Fig 5D’ and 5D”) and high amounts of Csk-YFP concentrated at the junction between muscles of adjacent abdominal segments. Next, we expressed Csk-YFP separately in muscles and neurons. By anti-GFP immunostaining, we saw that muscle-specific Csk-YFP protein spread throughout the muscle and clustered at the postsynaptic NMJ (Fig 5E–5E”) and muscle attachment sites (Fig 5E). By contrast, we only observed neuronally expressed Csk-YFP protein in the central nervous system (Fig 5F”). We did not observe neuronal-specific Csk-YFP protein at the presynaptic NMJ terminal (Fig 5F–5F”).

We further examined the synaptic localization of muscle expressed Csk-YFP by co-staining with monoclonal antibodies against the presynaptic ELKS/CAST active zone marker, Bruchpilot (Brp, Fig 5G), the GluRIIA glutamate receptor subunit (GluRIIA, Fig 5H), and the neural cell adhesion molecule Fasciclin II (FasII, Fig 5I). Not surprisingly, the domain of postsynaptic Csk-YFP staining was largely separate from the region of presynaptic Brp staining (Fig 5G). It also overlapped to only a minor degree with signal from the GluRIIA antibody (Fig 5H). Conversely, there was considerable colocalization between the Csk-YFP and FasII signals, although the two domains did not overlap completely (Fig 5I). These results suggest that Csk-YFP does not localize immediately at sites of synaptic transmission, but has a more periactive zone localization, similar to that previously documented for FasII [61].

We tested whether tissue specific expression of Csk-YFP could restore homeostatic plasticity to *GluRIIA; Csk* double mutant NMJs. Surprisingly, either pre- or postsynaptic expression of Csk-YFP was sufficient to restore homeostatic compensation (Fig 5J). By contrast, control recordings of *UAS-Csk-YFP; GluRIIA; Csk* NMJs lacking *GAL4* drivers still had completely blocked synaptic homeostasis (Fig 5J). Finally, despite baseline neurotransmission defects associated with Csk-YFP misexpression (Fig 5A–5C), synaptic homeostasis was robust when *UAS-Csk-YFP* was expressed both pre- and postsynaptically in the context of glutamate receptor loss (Fig 5K).

Our rescue experiments employed exogenous Csk-YFP. To probe the tissue specificity of endogenous Csk function, we knocked down *Csk* gene expression by RNAi. In the RNAi screen, when we knocked down *Csk* gene expression simultaneously in the pre- and postsynaptic compartments, synaptic homeostasis was completely blocked (Fig 1A). Here we found that postsynaptic knockdown alone partially attenuated synaptic homeostasis (Fig 5L). By contrast, presynaptic *Csk* knockdown alone left synaptic homeostasis intact (Fig 5L). Taken together the SFK overexpression experiments (Fig 4), and the tissue-specific Csk knockdown results suggest that postsynaptic (endogenous) Csk may be more important than presynaptic Csk in executing synaptic homeostasis. However, it is also true that exogenous Csk can support NMJ homeostatic plasticity when expressed either in the muscle or pan-neuronally.

### *Csk* mutant NMJs have dysregulated Fasciclin II expression

It is puzzling that Csk-YFP is sufficient to gate homeostatic plasticity from either nerve or muscle. One possibility is that Csk controls the release of a homeostatic factor into the synaptic cleft, and this factor is capable of supporting homeostatic function regardless of the tissue of origin. This hypothetical situation would be similar to that described for the secreted homeostatic factor Endostatin, which drives the induction of synaptic homeostasis when expressed either presynaptically or postsynaptically [19]. However, unlike Endostatin, Csk is not required for the rapid induction of synaptic homeostasis (Fig 1F). A second possibility is that Csk limits the release or expression of unknown NMJ factors that could dampen the NMJ homeostat.

**Fig 6:**
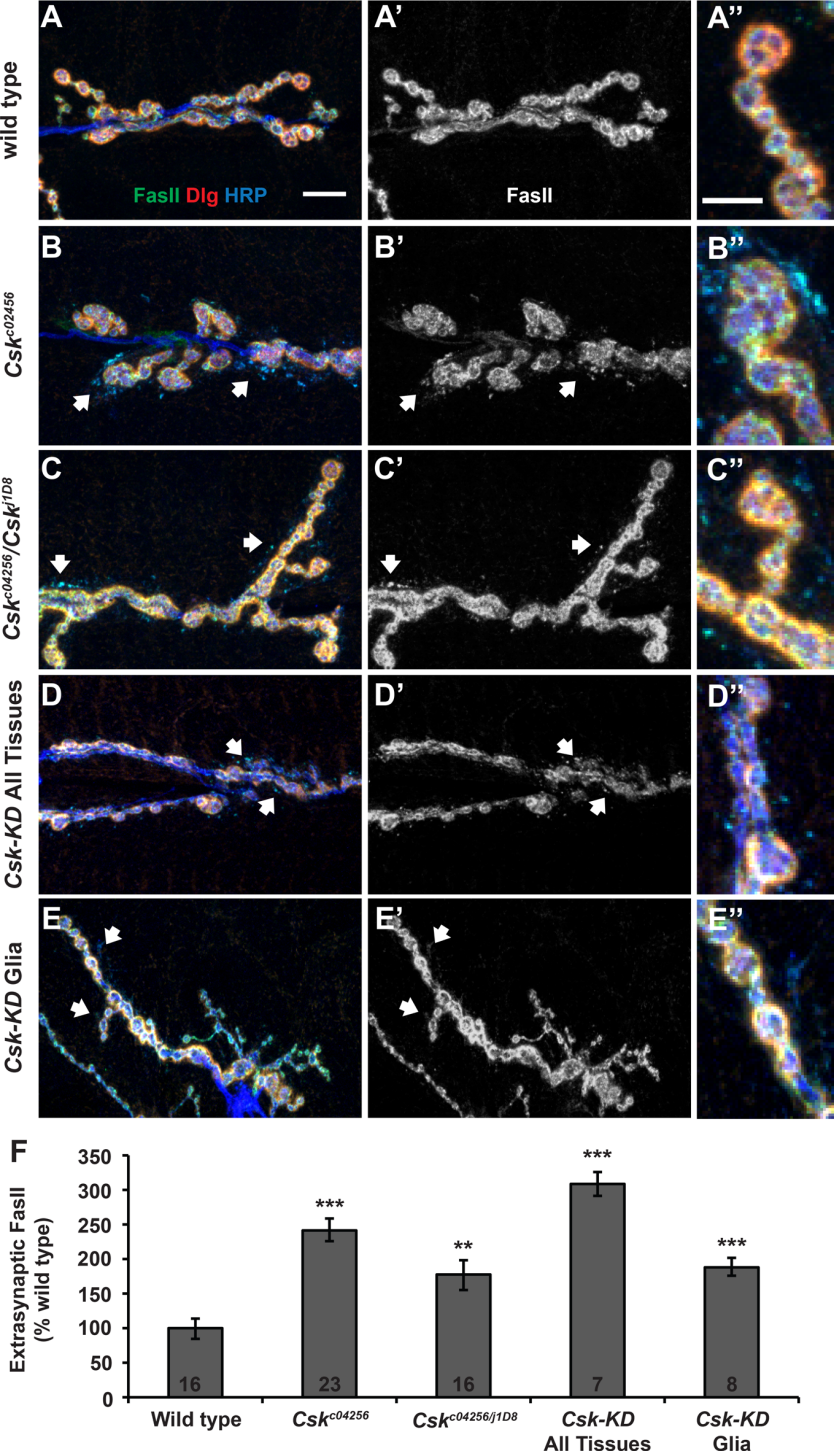
Glial Csk regulates Fasciclin II localization at the NMJ. **(A-E)** Immunostaining of anti-FasII (green), anti-Dlg (red), and anti-HRP (blue) at NMJs with the following genotypes: **(A-A”)** wild type, **(B-B”)** *Csk*^*c04256*^, **(C-C”)** *Csk*^*c04256*^*/Csk*^*j1D8*^, **(D-D”)** *Csk-RNAi* expressed in the whole animal (*Tubulin-Gal4*), and **(E-E”)** *Csk-RNAi* expressed only in glia (*Nrv2-Gal4*). Extra-synaptic FasII was defined as FasII signal found outside the Dlg-stained region. Areas with high levels of extra-synaptic FasII are indicated with white arrows. Scale bar = 10 μm for A and 5 μm for A”. **(F)** Relative levels of extra-synaptic FasII debris (extra-synaptic FasII staining area/total FasII staining area) present at the synapse. Values are represented as a percent of wild type to allow for appropriate comparison between multiple immunostaining experiments. For details on quantification of extra-synaptic FasII levels, see Methods. * *p* < 0.05, ** *p* < 0.01, *** *p* < 0.001 by Student’s T-test compared to wild type.

We attempted to identify NMJ proteins that could be responsive to levels of Csk activity. By immunostaining, we found that *Csk* mutant NMJ elaboration and bouton expansion were normal (Fig 2). However, we did note an abnormality: An anti-horseradish peroxidase (anti-HRP) antibody which marks neuronal membranes revealed a considerable amount of anti-HRP-positive staining beyond the normal nerve terminal domain at *Csk* mutant NMJs (Fig 6). Low-level debris shed from the presynaptic nerve is associated with normal synaptic growth processes [62]. By contrast, excess debris is thought to result from changes in synaptic activity and/or a failure of synaptic and cellular mechanisms to clear debris [62]. Additionally, the disruption of proteins that regulate synapse remodeling can induce small-diameter presynaptic membrane protrusions at the NMJ [63]. For *Csk* mutant NMJs, it was possible that the abnormal HRP-staining phenotype reflected aberrant synaptic activity, remodeling, or plasticity. Any of these parameters could be relevant to the homeostatic defects we had observed.

We conducted additional immunostaining. One molecule we considered for further study was the Neural Cell Adhesion Molecule (NCAM) ortholog Fasciclin II (FasII) because of the distinctive anti-HRP phenotype and partial co-localization with Csk-YFP (Fig 5I). FasII is one of the glycoproteins recognized by anti-HRP antibodies [64], and misexpression of FasII can cause abnormalities in synapse development [65]. FasII is present both pre- and postsynaptically; it forms homophilic interactions that span the synaptic cleft, and it is important for multiple forms of synaptic plasticity [66,67]. FasII levels at the NMJ are regulated by Mitogen-Activated Protein Kinase/Extracellular Signal-Regulated Kinase (MAPK/ERK) [68]. MAPK/ERK is a known target of SFK activity, providing a potential molecular link between Csk and FasII [69–72]. Finally, in vertebrate neurons, many cell adhesion molecules (CAMs) gate forms of homeostatic plasticity, possibly as transducers of trans-synaptic signals [73]. Taken together, these facts make FasII a candidate effector of homeostatic Csk activity.

We examined FasII protein at *Csk* mutant NMJs by immunofluorescence. We employed an anti-FasII monoclonal antibody, 1D4 (University of Iowa, Developmental Studies Hybridoma Bank) [74]. We also co-stained with anti-HRP and anti-Dlg. We used anti-Dlg staining to define the synapse boundary, and we observed and quantified “synaptic” and “extra-synaptic” anti-FasII staining. We defined staining that extended beyond the Dlg boundary to be extra-synaptic. At wild-type NMJs, extra-synaptic HRP-positive, FasII-positive puncta were present at low levels (Fig 6A and 6F), but in *Csk*^*c04256*^ and *Csk*^*c04256*^*/Csk*^*j1D8*^ NMJs, the relative proportion these extra-synaptic puncta was greatly increased (Fig 6B and 6C and 6F).

**Fig 7:**
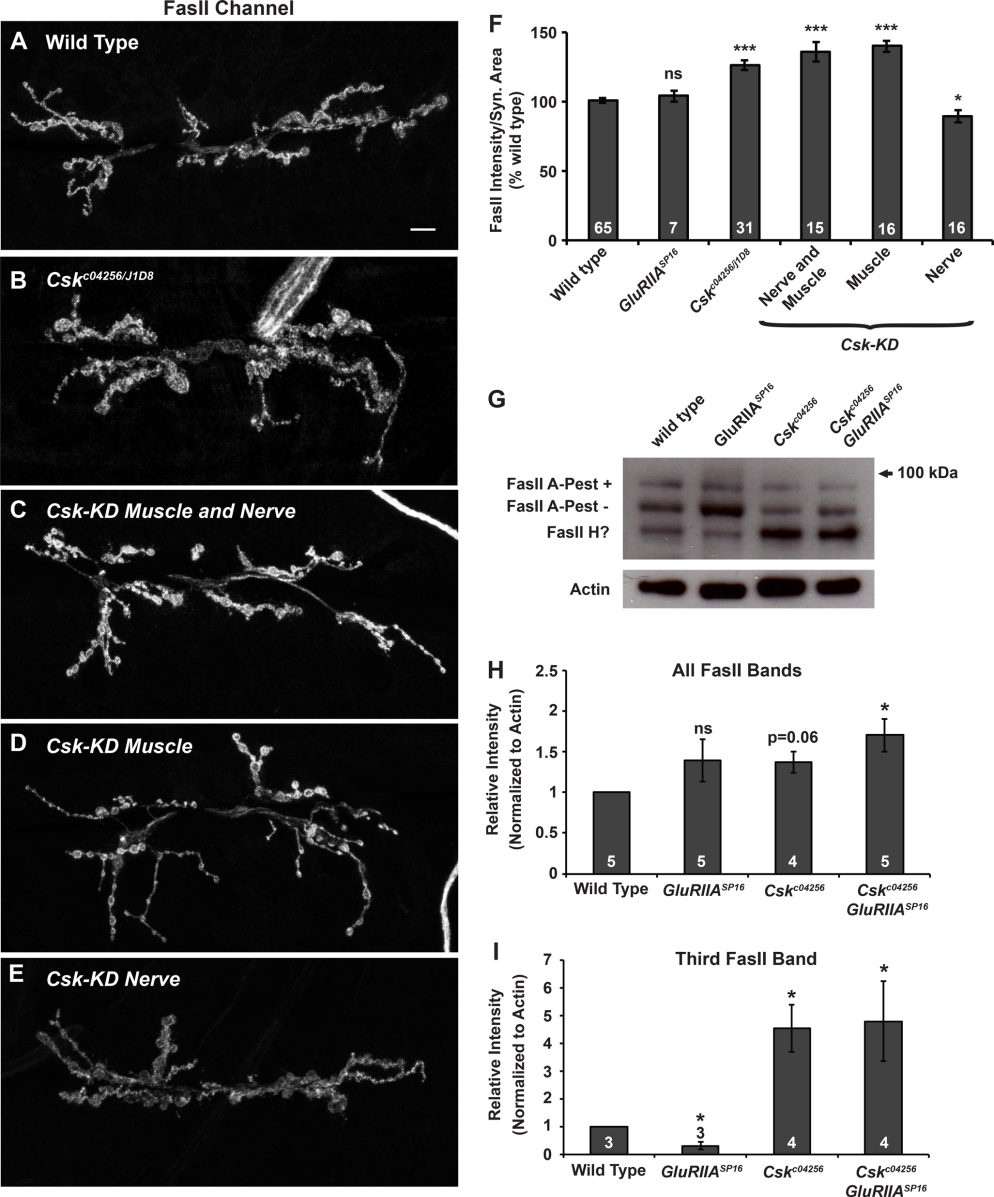
Expression of a Fasciclin II isoform is lowered during synaptic homeostasis and regulated by *Csk*. **(A-E)** Representative images of FasII immunostaining at NMJs that are **(A)** wild type, **(B)** *Csk*^*c04256*^*/Csk*^*j1D8*^, **(C)** expressing *Csk-RNAi* in muscle and neurons, **(D)** expressing *Csk-RNAi* in only muscle, *and* **(E)** expressing *Csk-RNAi* in only neurons. **(F)** Average values for synaptic FasII fluorescence intensity normalized to synapse area and normalized to wild type. **(G)** Western blot of FasII (DSHB 1D4 antibody) in protein extracts from whole third instar larvae. **(H, I)** Relative quantification of (H) total FasII intensity and (I) the intensity of the lowest molecular weight FasII band (the ‘third band’). Quantification in H and I is shown as a fold change relative to wild type. Values on/above bars indicate the number of biological replicates for each genotype. * *p* < 0.05, ** *p* < 0.01, *** *p* < 0.001, ns—not significant (*p* > 0.2) by Student’s T-test compared to wild type.

To check if this staining abnormality was specific to *Csk* gene function, we attempted to phenocopy it using RNAi. We observed excess extra-synaptic anti-FasII staining when we knocked down *Csk* function globally (*Tubulin-Gal4* >> *UAS-Csk[RNAi]*) (Fig 6D and 6F) or in the glia (*Nrv2-Gal4* >> *UAS-Csk[RNAi]*) (Fig 6E and 6F). Interestingly, we did not observe the same excess extra-synaptic anti-FasII staining when we knocked down *Csk* function either pre- or postsynaptically alone (Fig 7C–7E). The fact that the extra-synaptic anti-FasII pattern could be generated independent of neuronal or muscle knock down of *Csk* indicated that the extra-synaptic FasII staining phenotype itself was unlikely to be causal or predictive for impaired homeostatic signaling. Rather, it seemed to indicate a role for glial-derived Csk in FasII regulation and synapse development.

Synaptic contact sites are the loci most likely affected during homeostatic signaling. Therefore, we also quantified synaptic levels of FasII. *Csk*^*c04256*^*/Csk*^*j1D8*^ NMJs showed a statistically significant increase in synaptic FasII compared to wild-type controls (Fig 7A and 7B and 7F). Consistently, knock down of *Csk* by RNAi in the nerve and muscle or knockdown in muscle only also generated a significant increase in synaptic FasII (Fig 7C and 7D and 7F). By contrast, knock down of *Csk* in the nerve only caused a slight, but statistically significant decrease in synaptic FasII levels (Fig 7E and 7I). In sum, *Csk* genetic manipulations that impair or partially impair homeostatic plasticity also result in statistically significant increases in anti-FasII staining at the NMJ.

We turned to biochemistry to test if we could identify differences in the expression of individual FasII isoforms in *Csk* mutants. There exist multiple isoforms of FasII. We generated larval lysates from various genetic backgrounds, ran the lysates on a gradient protein gel, and conducted Western blots using the 1D4 anti-FasII monoclonal antibody. This antibody recognizes known FasII isoforms that contain an intracellular domain, including FasII A-PEST+ (full) and FasII A-PEST- (29-aa exon excluded) [75].

For wild-type lysates, we detected two strong FasII A bands, which migrated as predicted for the PEST+ and PEST- 1D4-reactive isoforms (Fig 7G) [75]. The gradient gel also revealed a third, previously unreported 1D4-reactive band at a slightly lower molecular weight (Fig 7G). This band could correspond to a poorly characterized, predicted FasII isoform containing an intracellular domain (FasII H, Accession AHN59326.1). It could also be a FasII A degradation product or a non-specific band. We quantified the relative intensity of the 1D4-reactive bands for lysates of four genotypes: 1) wild type, 2) *GluRIIA*^*SP16*^, 3) *Csk*^*c04256*^, and 4) *GluRIIA*^*SP16*^*; Csk*^*c04256*^ (Fig 7G–7I). Surprisingly, the intensity of the low molecular weight band was significantly reduced in *GluRIIA*^*SP16*^ mutant (homeostatically competent) lysates (Fig 7G and 7I). By contrast, in *Csk* mutant lysates or *GluRIIA; Csk* mutant (homeostatically incompetent) lysates, the low molecular weight FasII band became quite strong, increasing in intensity by > 300%, normalized to lysate actin levels (Fig 7G and 7I). These experiments demonstrate that synaptic FasII levels and isoform-specific levels are responsive to genetic conditions (*GluRIIA* and *Csk* mutations) that alter the homeostatic capacity of the NMJ.

### Misexpression of FasII impairs synaptic homeostasis—loss of FasII does not

At the NMJ, increased synaptic FasII could contribute to the impairment of homeostatic plasticity. Alternatively, synaptic FasII could have little or no influence on homeostatic plasticity and simply be acting as a secondary biological reporter of Csk-mediated signaling processes. We acquired *FasII* mutant and overexpression genetic tools to test these ideas.

**Fig 8:**
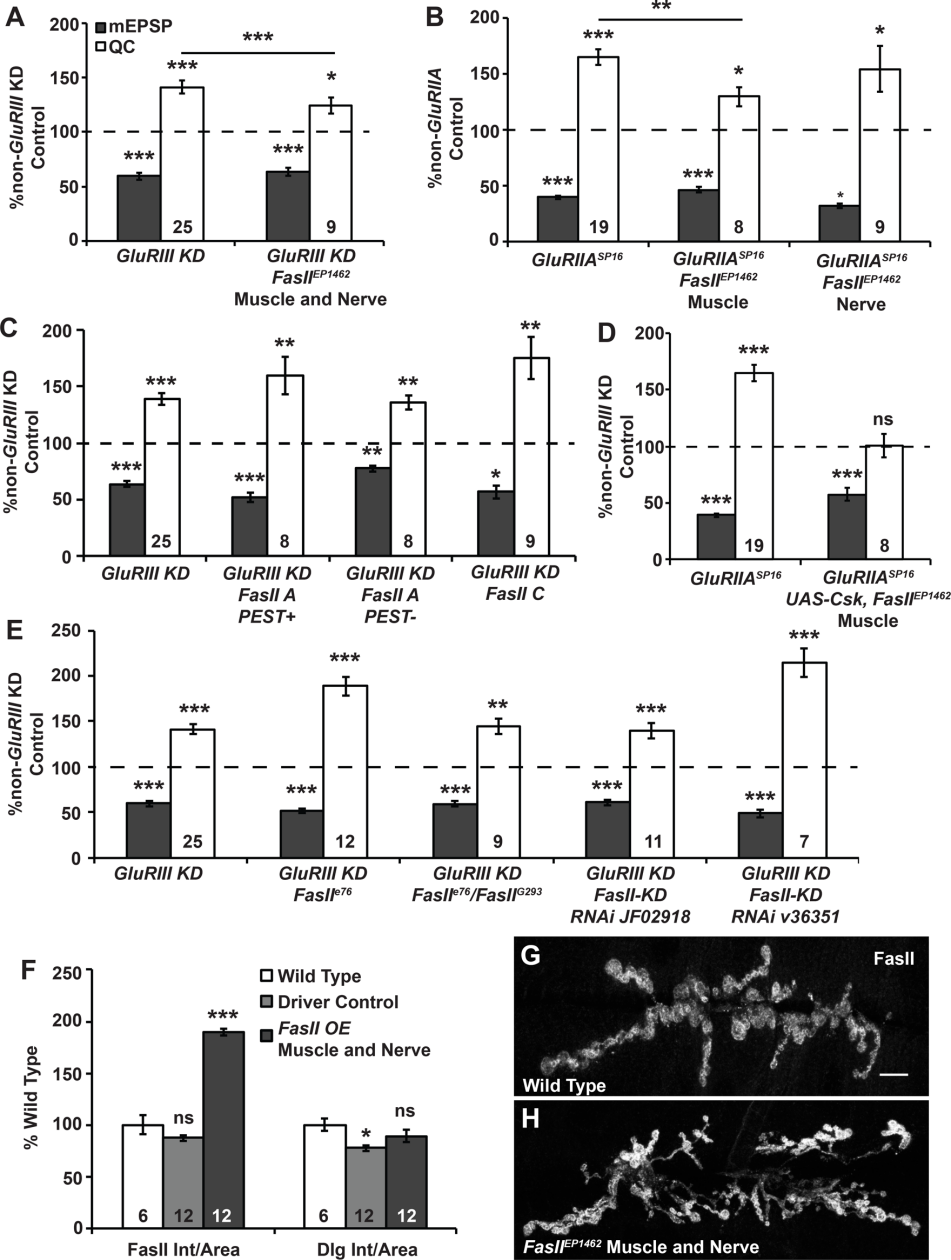
Excess FasII impairs synaptic homeostasis. **(A-E)** Values for mEPSP amplitude (gray) and quantal content (QC; white) normalized to genetic controls (dashed line) that lack a homeostatic challenge (non-*GluRIII KD* or non-*GluRIIA* controls). **(A)** Trans-synaptic FasII overexpression (O/E) from the FasII endogenous locus (*FasII*^*EP1462*^) shows partial impairment of synaptic homeostasis, as does **(B)** muscle-specific overexpression with *FasII*^*EP1462*^. **(C)** Overexpressing specific isoforms of FasII does not impair homeostatic compensation. **(D)** Overexpressing Csk-YFP in addition to FasII^EP1462^ fails to suppress the homeostatic defects of FasII O/E seen in B. **(E)** Neither *FasII* loss-of-function mutations nor *FasII* knockdown (KD) impairs synaptic homeostasis. **(F)** Average values for synaptic FasII and Dlg fluorescence intensity normalized to synapse area. **(G-H)** Representative images of FasII immunostaining for trans-synaptic FasII overexpression. Scale bar = 10 μm. * *p* < 0.05, ** *p* < 0.01, *** *p* < 0.001, ns—not significant (*p* > 0.08) by Student’s T-test comparing homeostatically challenged mutants to their unchallenged (non-*GluRIIA*) controls.

The *FasII* transposon insertion allele, *FasII*^*EP1462*^, is located upstream of the *FasII* translation start site [76]. This transposon contains a *UAS* sequence that can be exploited to overexpress *FasII* from its endogenous locus [76]. We overexpressed *FasII* at the NMJ by crossing *FasII*^*EP1462*^ to a Drosophila stock with pre- and postsynaptic *GAL4* drivers and a *UAS-GluRIII[RNAi]* construct to induce a homeostatic challenge [33] (Fig 8). This cross resulted in an 89.5 ± 3.4% increase in NMJ FasII levels per unit synapse area relative to wild type (*p* < 0.001, Student’s T-Test; Fig 8F–8H). It also resulted in a significant impairment of homeostatic compensation (Fig 8A)–but not a complete impairment. Next, we employed *FasII*^*EP1462*^ to overexpress FasII in a tissue-specific manner. Neither presynaptic overexpression of FasII alone, nor postsynaptic overexpression of FasII alone erased synaptic homeostasis in a *GluRIIA* mutant background (Fig 8B). However, postsynaptic overexpression did cause a partial impairment of synaptic homeostasis that was quantitatively similar to dual-tissue overexpression (Fig 8B). These data are consistent with the possibility that excess postsynaptic FasII is sufficient to impair synaptic homeostasis.

We tested if overexpression of individual FasII isoforms could recapitulate the homeostatic defects we observed with overexpression of the entire *FasII* locus. We acquired three FasII expression transgenes encoding the most well-characterized isoforms: *UAS-FasII-A PEST+*, *UAS-FasII-A PEST-*, and *UAS-FasII-C* [75]. The two FasII-A isoforms contain extracellular and cytoplasmic domains, and the FasII-C isoform is strictly extracellular and thought to be bound to the membrane by glycosylphosphatidylinositol (GPI) linkage [77]. We utilized the transgenes to individually misexpress these three isoforms of FasII in the context of a *GluRIII[RNAi]* knockdown. Unlike overexpression from the endogenous gene locus using *FasII*^*EP1462*^, none of these single misexpression experiments recapitulated the homeostatic impairment (Fig 8C). This result could mean that overexpression of multiple isoforms is needed to block synaptic homeostasis, or it could mean that a different, less well-characterized isoform (possibly FasII-H) is responsible for the block in synaptic homeostasis.

Csk loss caused dysregulated synaptic FasII (Figs 6–7), suggesting that FasII may reside downstream of Csk. To characterize the regulatory relationship of Csk and FasII further, we raised Csk levels in the muscle and then tested if this manipulation could ameliorate the defect in synaptic homeostasis caused by *FasII*^*EP14162*^ overexpression. However, we found that when we overexpressed both *UAS-Csk-YFP* and *FasII*^*EP14162*^ in a *GluRIIA*^*SP16*^ mutant background, synaptic homeostasis was still impaired (Fig 8D). Our collective data suggest that FasII function resides downstream of Csk function, and excess FasII can overwhelm the ability of Csk to promote synaptic homeostasis (Figs 6–8).

Next we tested if *FasII* loss-of-function conditions could also cause defects in synaptic homeostasis. Null or strong loss-of-function alleles like *FasII*^*G0293*^ [78] show lethality at various stages of development, whereas *FasII*^*e76*^ is a semi-viable, partial loss-of-function mutation [67]. In a *GluRIII[RNAi]* knockdown background, we examined the electrophysiological effects of the *FasII*^*e76*^ mutant allele as a hemizygote (*FasII*^*e76*^*/Y* males) or in combination with the *FasII*^*G0293*^ allele (*FasII*^*e76*^*/FasII*^*G0293*^ females). We also tried knocking down FasII function using RNAi lines, *FasII*^*GD14486*^ (Vienna Drosophila Resource Center line, v36351) and *FasII*^*JF02918*^ (Bloomington Drosophila Stock Center TRiP collection). None of these *FasII* loss-of-function conditions impaired homeostatic compensation (Fig 8E). The most plausible interpretation is that synaptic homeostasis is sensitive to elevated levels of FasII, but not to diminished levels of FasII. Alternatively, the low levels of FasII present at hypomorphic NMJs may be sufficient for homeostatic compensation to occur.

Misexpression of FasII could disrupt NMJ development and cause homeostatic defects as a consequence of aberrant NMJ growth. Overexpression of FasII in neurons has been reported to result in axon pathfinding defects, ectopic synapses at some sites, and reduced or abnormal synapses at other sites [76]. Furthermore, concurrent pre- and postsynaptic overexpression of the GPI-linked form of FasII has been reported to induce the production of satellite boutons [79], and misexpression of FasII in muscle fibers can alter NMJ innervation patterns [65]. Therefore, we needed to determine if the FasII overexpression conditions we used to impair synaptic homeostasis also triggered gross abnormalities in NMJ development. Compared to wild-type synapses, we found no significant changes in bouton number or muscle area when *FasII*^*EP1462*^ was overexpressed from its endogenous locus during a *GluRIII[RNAi]*-induced homeostatic challenge (S1D–S1F Fig). This result supports the idea that the homeostatic defects associated with FasII overexpression are not caused by gross NMJ undergrowth.

### Reducing FasII levels in *Csk* mutants restores homeostatic compensation

We have shown that the *Csk* mutant NMJs have altered FasII NMJ expression (Figs 6 and 7). Additionally, misexpression of FasII partially impairs synaptic homeostasis (Fig 8); loss of FasII leaves synaptic homeostasis intact (Fig 8). Therefore, we tested whether reducing FasII levels could restore homeostatic compensation to *GluRIIA; Csk* mutant NMJs.

**Fig 9:**
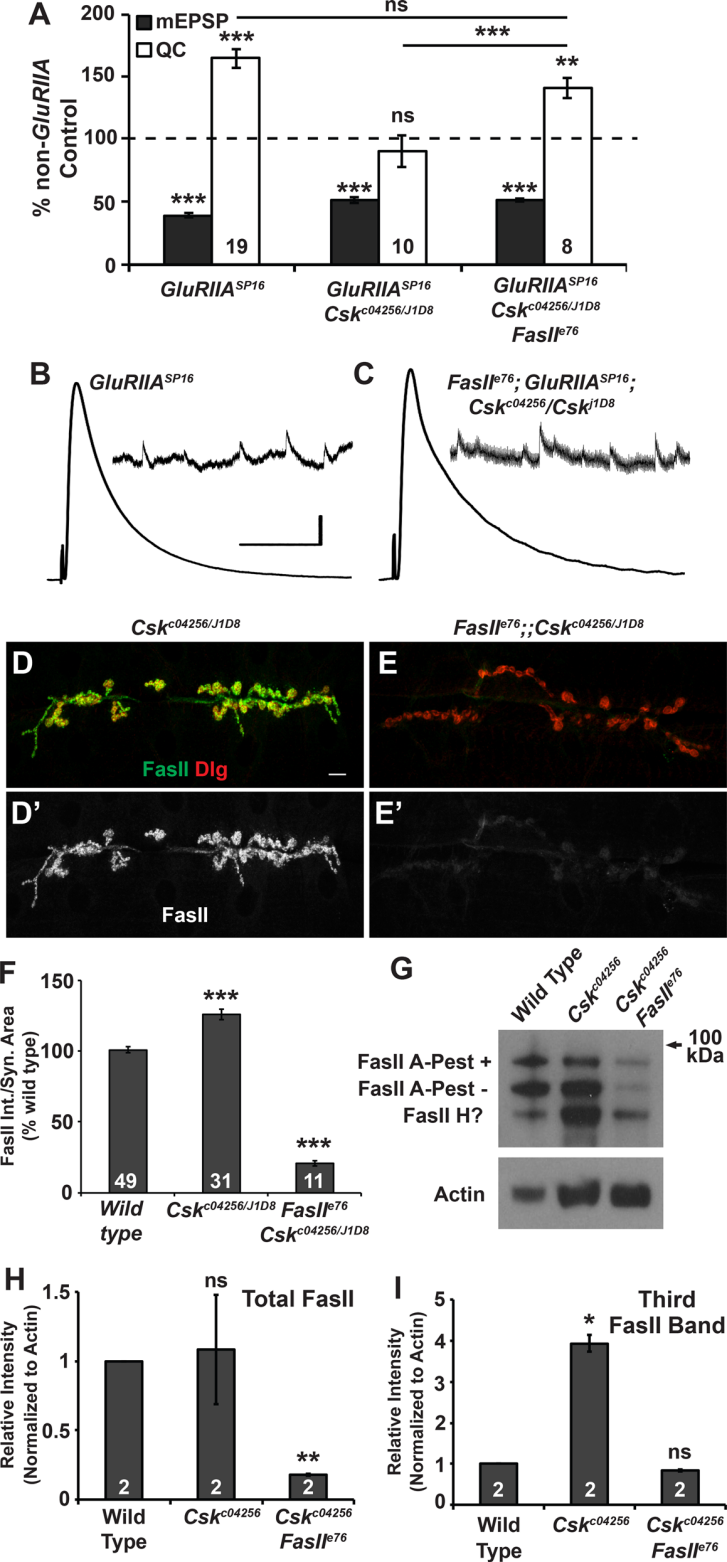
Reducing FasII levels in *GluRIIA; Csk* mutants restores homeostatic compensation. **A)** Values for mEPSP amplitude (gray) and quantal content (QC; white) normalized to genetic controls (dashed line) that lack a homeostatic challenge. *FasII*; *GluRIIA*; *Csk* triple mutants have intact homeostatic plasticity. **(B-C)** Representative electrophysiological traces for data shown in A. Scale bar for EPSP (mEPSP) traces: y = 5 mV (0.5 mV), x = 50 ms (1 s). **(D-E’)** Representative images of NMJs stained for FasII (green, gray channel) and Dlg (red) for (D, D’) *Csk* mutant and (E, E’) *FasII*, *Csk* double mutant NMJs. Scale bar = 10 μm. **(F)** Quantification of FasII staining intensities per synapse area for the genotypes shown in D-E’. **(G)** Western blot of FasII (DSHB 1D4 antibody) in protein extracts from whole third instar larvae. **(H, I)** Relative quantification of (H) total FasII intensity and (I) the intensity of the lowest molecular weight FasII band (the ‘third band’). Values are fold changes relative to wild type. * *p* < 0.05, ** *p* < 0.01, *** *p* < 0.001, ns—not significant (*p* > 0.4) by Student’s T-test and for the bars in A, which are *p*-values from ANOVA (Tukey’s post-hoc) comparison between the three genotypes shown.

*GluRIIA; Csk* double mutants had completely blocked synaptic homeostasis (Figs 1 and 9A), but remarkably, synaptic homeostasis was restored in the *FasII*^*e76*^*/Y*; *GluRIIA*^*SP16*^; *Csk*^*c04256/J1D8*^ triple loss-of-function mutant (Fig 9A–9C). This result suggested that homeostatic defects observed at *GluRIIA; Csk* mutant NMJs could be due to synaptic FasII misexpression. To verify that the *FasII*^*e76*^ genetic manipulation employed in our electrophysiological analyses depressed FasII protein expression, we conducted immunostaining and Western blotting. *FasII*^*e76*^ markedly reduced synaptic FasII protein (Fig 9D–9F), and it also muted the expression of all 1D4-reactive FasII bands on Western blots, including the low molecular weight band that is enhanced in *Csk* loss-of-function mutants (Fig 9G–9I).

Finally, we assessed NMJ growth in *FasII*; *Csk* double mutants by counting synaptic boutons and measuring muscle size. Absolute bouton number was unchanged relative to wild type (S1A Fig). However, due to a small decrease in muscle size, bouton number per unit muscle area was increased for segment A2, synapse 6/7 (S1B and S1C Fig). This change persisted whether there was a *GluRIIA*^*SP16*^ homeostatic challenge or not (S1C Fig). Together, our data show that *FasII* loss-of-function mutations can suppress the defects in synaptic homeostatic plasticity caused by *Csk* mutations. There may be a slight developmental growth component that correlates with this electrophysiological suppression.

## Discussion

Here we identify C-terminal Src Kinase (Csk) as a regulator of sustained homeostatic plasticity at the Drosophila NMJ (Fig 1). Additionally, we show that synaptic homeostasis is impaired when the activities of Csk targets, the SFKs Src42A and Src64B, are increased in the postsynaptic muscle (Fig 4). Interestingly, either presynaptic or postsynaptic Csk is sufficient to support the homeostatic capabilities of the NMJ (Fig 5). This may indicate that Csk controls cellular release or synaptic expression of factors that impact homeostatic signaling. In this vein, the trans-synaptic NCAM homolog FasII appears to be a negative homeostatic factor regulated by Csk in the postsynaptic muscle (Figs 6–8). Notably, loss of *FasII* gene function restores homeostatic capacity to *GluRIIA; Csk* mutant NMJs (Fig 9).

### Csk and postsynaptic homeostatic signaling molecules

While Csk-YFP expression was sufficient to restore homeostatic compensation either pre- or postsynaptically, we favor a model in which postsynaptic Csk plays the predominant role in synaptic homeostasis. This is supported by several observations. First, Csk-YFP was only detected at the NMJ when postsynaptically expressed (Fig 5). Additionally, when the endogenous function of Csk was examined by RNAi-meditated knockdown, only postsynaptic *Csk* knockdown led to impaired synaptic homeostasis (Fig 5) and increased FasII levels (Fig 7). Additionally, postsynaptic FasII overexpression was sufficient to impair homeostatic plasticity (Fig 8). These combined data suggest that, in the case of FasII-mediated homeostatic processes, postsynaptic Csk is the primary player in synaptic homeostasis. The ability of presynaptic Csk-YFP expression to restore homeostatic competency indicates that presynaptic Csk may also play a role in these processes, likely through a FasII-independent pathway.

The identification of homeostatic functions for Csk and SFKs in the muscle expands our understanding of the postsynaptic proteins that regulate Drosophila NMJ neurotransmission through retrograde signaling. A small number of postsynaptic factors have been previously implicated in this process, including the protein scaffold Dystrophin (Dys) [80,81], the RhoGAP Crossveinless-c (Cv-c) [80], the nuclear import factor Importin-13 (Imp13) [82], Calcium/Calmodulin protein Kinase II (CaMKII) [83], Drosophila target of rapamycin (TOR) [32], and S6 Kinase (S6K) [32]. In the cases of Dys, Cv-c, and Imp13, loss of any of these factors in the muscle causes a *de novo* increase of presynaptic neurotransmitter release [80–82]. This likely means that they are negative regulators of retrograde signaling. However, these factors remain to be fully characterized in the context of a challenge to synapse function, such as glutamate receptor loss. In the cases of TOR and its phosphorylation target, S6K, loss-of-function mutations block the long-term maintenance of homeostatic compensation [32], and postsynaptic overexpression or activation of these factors instructively induces *de novo* increases in presynaptic quantal content [32].

As a homeostatic signaling molecule, Csk appears to be distinct from these previously described factors. One difference between TOR/S6K and Csk is that elevated Csk levels do not induce a *de novo* increase in quantal content (Fig 5C). Moreover, transgenic Csk-YFP only induces increases in vesicle release in the context of a homeostatic challenge, like glutamate receptor loss (Fig 5K). Another difference is that Csk is capable of conferring homeostatic competence to the NMJ from either the nerve or the muscle (Fig 5J), while factors like TOR and S6K perform their homeostatic functions from the muscle [32]. This result suggests that Csk could be more generally involved in regulating the expression or localization of factors that influence homeostatic plasticity over extended periods of developmental time.

One unresolved issue is how exogenous Csk-YFP was able to restore homeostatic capacity to *Csk* mutant NMJs. It is possible that neuronal Csk and SFKs regulate unknown cellular targets that drive the presynaptic expression of homeostatic plasticity. Some avenues for future experiments are suggested by prior studies. For example, we know from vertebrate neuronal preparations that SFKs are capable of regulating neurite outgrowth, cell shape and cell growth by phosphorylating the guanine exchange factor, Ephexin (Exn) [84–87]. At the NMJ, we have previously demonstrated that presynaptic Exn supports the long-term maintenance of homeostatic plasticity in conjunction with Ca_V_2-type calcium channels [30]. Therefore, it is possible that presynaptic Csk can regulate synaptic homeostasis via Exn regulation.

Another possible presynaptic Csk/SFK target is the vesicle-associated protein Synapsin (Syn). In rodent neurons, Src phosphorylation of Syn diminishes the readily releasable pool (RRP) of synaptic vesicles, and expression of a non-phosphorylatable version of Synapsin (Y301F) increases the size of the RRP [88,89]. Since RRP size increase is a key determinant in homeostatic compensation at the Drosophila NMJ [20,23], *Csk* mutations might lead to excessive SFK-mediated phosphorylation of Drosophila Syn and a failure to increase RRP size and QC when the synapse is homeostatically challenged. Such a model could explain our finding that *GluRIIA; Csk* double mutant NMJs do not deplete evoked responses to the same degree as *GluRIIA* mutant NMJs when challenged with a high frequency stimulus train (Fig 3F–3G).

### Csk and the long-term maintenance of synaptic homeostasis

We know very little about how synapses maintain normal levels of function for extended periods of developmental time. When considering the Drosophila NMJ, it is helpful to compare and contrast the long-term maintenance of synaptic homeostasis with its acute induction. Induction can occur on a timescale of minutes; it does not require protein translation, and it occurs even when the presynaptic nerve is severed from the neuronal soma [10]. By contrast, the long-term maintenance of synaptic homeostasis requires protein translation-activating factors in the muscle (TOR/S6K) [32], cytoplasmic signaling molecules in the neuron (the Rho-GEF Ephexin) [30], and a neuronal transcription factor (Gooseberry) [31]. These factors seem to act in concert to consolidate increases in QC in response to a homeostatic challenge. To this short list, we add C-terminal Src kinase, which can support homeostatic compensation from either the neuron or the muscle. We also add the misexpression of Src42A, Src64B, and FasII as factors that impair homeostatic outputs. One caveat to our conclusion that Csk acts as a factor that controls only the long-term consolidation of synaptic homeostasis is that the *Csk* mutant conditions used for this study were not null. *Csk* null mutants arrest well before the third instar larval stage. Therefore, our data cannot formally rule out a role for Csk in the acute induction of homeostatic plasticity.

How synapses work to stabilize outputs for extended periods of developmental time could be relevant for understanding neurological disorders that manifest after decades of life. An interesting example that has direct parallels to our experimental system is the neuromuscular disorder myasthenia gravis (MG). Autoimmune inhibition of human NMJ acetylcholine receptors (AChRs) is one cause of MG. In a phenomenon that is similar to the homeostatically challenged Drosophila NMJ, electrophysiological recordings from myasthenic muscle show that these NMJs have a reduced quantal size and a compensatory increase in QC [90,91]. It is possible that the cellular events that drive the increase in QC at myasthenic NMJs serve to quell disease manifestations until later in life. Interestingly, the NCAM CD56 has been shown to be decreased in cells treated with corticosteroids, which is a common method to combat MG [92].

### FasII and Cell Adhesion Molecules (CAMs) as modulators of homeostatic plasticity

FasII forms homophilic trans-synaptic complexes [66,67,77]. Therefore, FasII is an intriguing candidate to modulate retrograde signaling at the NMJ. To date, examples of CAMs (or CAM complexes) that have been implicated in synaptic homeostasis include N-cadherin/β-catenin [93,94], β3 integrins [95,96], Ephrin ligands/Eph receptors [30,97,98], class I major histocompatibility complex proteins (MHC-1) [99], and α-neurexins [100]. Some of these CAMs are hypothesized to serve as scaffolds that draw other molecules to the synapse; in turn, the recruited molecules could modulate neurotransmission. [73].

How might synaptic FasII/NCAM impinge upon homeostatic signaling in the context of *Csk* loss? Prior work has demonstrated that pre- and postsynaptic FasII can exert effects on synaptic stabilization [67]. Temporary increases in muscle FasII in particular can result in novel synaptic contacts that are stable [65]. In principle, it is possible that our *Csk* and *FasII* genetic manipulations spurred a small number of inappropriate synaptic connections that occluded the homeostatic capacity of the NMJ.

A second potential explanation for our data is that FasII impairs the function of a known homeostatic regulator. At the NMJ, the majority of identified homeostatic factors function in the presynaptic neuron [101]. These include regulators of presynaptic vesicle release, ion channels, cytoplasmic signaling molecules, and key components of the active zone [101]. Among these factors, one potential target of FasII/NCAM regulation is the Drosophila Eph receptor (Eph) [30], upstream of Exn. Compelling parallels exist between Drosophila and mammalian data regarding the function of Eph receptors in synaptic plasticity. Mammalian NCAM physically interacts with EphA Receptors (EphAs) [102,103], and an NCAM/EphA interaction was recently reported to dictate excitatory/inhibitory balance in the mouse prefrontal cortex [103]. At the Drosophila NMJ, it was previously reported that the Eph receptor functions in conjunction with the cytoplasmic guanine exchange factor Exn and Ca_V_2-type calcium channels to homeostatically modulate presynaptic neurotransmitter release [30]. Since Eph and Exn also dictate the long-term maintenance of NMJ homeostasis [30], this opens the possibility that trans-synaptic FasII acts as a modulator of Eph.

Other retrograde factors proposed to function in the synaptic cleft for homeostatic compensation are the BMP ligand Glass Bottom Boat (Gbb) [15,104] and the Drosophila homolog of Endostatin [19]. Endostatin is produced when the Collagen XV/XVIII homology factor Multiplexin is cleaved by matrix-metalloproteinases (MMPs) or cysteine cathepsins [105]. Interestingly, in mammalian systems, increased NCAM expression leads to a decrease in the secretion of several MMPs [106]. This relationship has not been examined directly in Drosophila, but FasII and MMPs are both required for proper motor neuron fasciculation, axon guidance, and target recognition [107–112], suggesting that a regulatory relationship between these molecules may be present in Drosophila as well. Future experiments will be needed to determine precisely how increased FasII expression at the NMJ results in reduced homeostatic capacity.

## Materials and Methods

### Drosophila stocks

Drosophila stocks carrying various mutant alleles, *GAL4* drivers, or *UAS*-driven transgenes were used for this study. Stocks were either obtained from the Bloomington Drosophila Stock Center (BDSC, Bloomington, IN), the Vienna Drosophila RNAi Center (VDRC, Vienna, Austria), or from researchers who generated them. Mutant alleles include: *Csk*^*c04256*^ [36], *Csk*^*j1D8*^ [39], *Src42A*^*k10108*^ [59], *FasII*^*EP1462*^ [76], *FasII*^*G0293*^ [78], *FasII*^*e76*^ [77], *Src64B*^*KO*^ [113]. *GAL4* drivers include: *elaV(C155)-GAL4* [114], *OK371-GAL4* [107,115], *Scabrous-GAL4* [43], *BG57-GAL4* [43], *MHC-GAL4* [67], *MyoD-GAL4* [116]. *UAS* transgenes include: *UAS-Src64B*^*UY1332*^ [54], *UAS-Src64B*.*C* (J.M. Dura and J. Cooper donation to BDSC), *UAS-Src42A*^*YF*^ [37], *UAS-Src42A*^*WT*^ [37]. *UAS-RNAi* lines from VDRC [117] include: *UAS-Csk[RNAi]* (VDRC# 32877, *Csk*^*GD9345*^) and *UAS-FasII[RNAi]* (VDRC# 36351, *FasII*^*GD14486*^). UAS-RNAi lines from BDSC include: *UAS-FasII[RNAi]* (*FasII*^*JF02918*^). We constructed *UAS-GluRIII[RNAi]* [33]. To construct *UAS-Csk-YFP* transgenic lines, we PCR amplified the PH isoform of *Csk* from cDNA clone LP09923 from the Drosophila Genomics Resource Center (DGRC, Bloomington, IN), and subsequently cloned the amplicon into a pUAST vector with a C-terminal YFP tag, using the Drosophila Gateway Vector Collection (T. Murphy, Carnegie Institute of Washington). Transgenic animals were made by injecting DNA into embryos using standard procedures (BestGene, Inc., Chino Hills, CA).

### Drosophila husbandry

Fruit flies were reared in chambers with temperature control; experimental and control animals were raised in parallel under identical conditions. We conducted *UAS-Src64B* and *UAS-Src42A* (*UAS-SFK*) overexpression experiments by using *elaV(C155)-GAL4* or *OK371-GAL4* for presynaptic expression and *BG57-GAL4* or *MHC-GAL4* for postsynaptic expression. In all cases for *UAS-SFK* expression, the conditions were lethal when animals were raised at 29°C, but lowering the rearing temperature was sufficient to circumvent lethality and allow for electrophysiological analyses. The specific *UAS-SFK/GAL4* combinations and rearing temperatures that we used for analysis are found in S1 Table.

### Electrophysiology and analysis

Wandering third instar larvae were selected for electrophysiological recordings. Sharp electrode recordings were taken from muscle 6 of abdominal segments 2 and 3, as previously described [10,30,118]. Larvae were dissected in a modified HL3 saline with the following components (and concentrations): NaCl (70 mM), KCl (5 mM), MgCl2 (10 mM), NaHCO3 (10 mM), sucrose (115 mM = 3.9%), trehalose (4.2 mM = 0.16%), HEPES (5.0 mM = 0.12%), and CaCl_2_ (0.5 mM, unless otherwise indicated). Data were collected using Axopatch 200B or Axoclamp 900A amplifiers (Molecular Devices, Sunnyvale, CA), digitized using a Digidata 1440A data acquisition system (Molecular Devices), and recorded with pCLAMP 10 acquisition software (Molecular Devices). For presynaptic nerve stimulation, a Master-8 pulse stimulator (A.M.P. Instruments, Jerusalem, Israel) and an ISO-Flex isolation unit (A.M.P. Instruments) were utilized to deliver 1 ms suprathreshold stimuli to the appropriate segmental nerve. The average spontaneous miniature excitatory postsynaptic potential (mEPSP) amplitude was quantified by measuring the amplitude of approximately 100–200 individual spontaneous release events per NMJ (Mini Analysis, Synaptosoft, Fort Lee, NJ). The average per-NMJ mEPSP amplitudes were then averaged for each genotype. The average evoked EPSP amplitude was calculated for each NMJ. Quantal content was determined for each recording by calculating the ratio of average EPSP and average mEPSP amplitudes. Quantal contents were calculated for each recording and then averaged across NMJs of the indicated genotypes. Where indicated in S1 Table, quantal contents were corrected for non-linear summation as described [119]. Long trains of stimuli were analyzed using an auto-analyze function in Mini Analysis.

### Immunostaining

Third instar larvae were filleted in HL3 saline. Dissected animals were fixed for 3 minutes in Bouin’s fixative (Ricca Chemical Company, Arlington, TX), washed using standard procedures, and incubated in primary antibodies at room temperature for two hours. This was followed by additional washes and another two-hour incubation in secondary antibody at room temperature. Staining was performed using the following primary antibodies: mouse anti-Synapsin (3C11) 1:50 [42] (Developmental Studies Hybridoma Bank, University of Iowa–DSHB – deposited by Buchner, E.); rabbit anti-Dlg 1:30,000 [43]; mouse anti-Brp (nc82) 1:250 [44] (deposited to DSHB by Buchner, E.); mouse anti-FasII (1D4) 1:900 [74] (deposited to DSHB by Goodman, C.); mouse anti-GluRIIA (8B4D2) 1:500 [120] (deposited to DSHB by Goodman, C.). The following fluorophore-conjugated antibodies were also used (Jackson ImmunoResearch Laboratories, Inc., West Grove, PA): goat anti-mouse-488 1:1000 (DyLight); goat anti-rabbit-549 1:2000 (Dylight); goat anti-HRP-TRITC 1:1000, Alexa-647 goat anti-HRP 1:500. Larval preparations were mounted in Vectashield (Vector Laboratories, Burlingame, CA) and imaged at room temperature using Zen software on a Zeiss 700 LSM mounted on an Axio Observer.Z1 using an EC Plan-Neofluar 40X Oil DIC Objective (aperture 1.30) or an EC Plan-Apochromat 63x Oil DIC Objective (aperture 1.40) (Carl Zeiss Microscopy, Jena, Germany). For each experiment, experimental and control larval preps were stained in the same container, mounted on the same slide, imaged using identical acquisition settings, and analyzed using the same procedure and thresholds.

### Image analysis: Assessing synaptic morphology and Fasciclin II quantification

Bouton and active zone numbers were quantified semi-automatically using the ‘Spots’ function in Imaris x64 v7.6.0 (Bitplane, Zurich Switzerland). Boutons were counted using the anti-Synapsin channel with the XY diameter set at 3 μm. Active Zones were counted using the anti-Brp channel with an XY diameter of 0.3 μm. The threshold was adjusted so that each bouton/active zone was recognized once. Any errors in automated counting were corrected by hand to arrive at the final value. FasII levels and localization were assessed using ImageJ 1.48s/Java 1.6.0_24 (64-bit) with Fiji plugins. FasII levels were assessed as follows: Z-stack images were compressed using the maximum projection function; regions of interest (ROIs) were hand drawn to exclude any non-synaptic structures in the image; a minimum threshold was set for each channel to eliminate background fluorescence and held consistent within each experiment; the Measure function was used to assess fluorescence intensity and area for each channel (FasII(488), Dlg(549), HRP(647)); total FasII and Dlg intensities were normalized to the synaptic area of the HRP channel to control for variation in synaptic size. Extra-synaptic FasII levels were determined as follows: Z-stack images were compressed using the maximum projection function; a mask was generated based on the Dlg channel; this mask was used to generate ROIs that defined the synaptic area and the extra-synaptic area; a third ROI was hand drawn to exclude any non-synaptic structures in the image; this third ROI was combined with the DLG mask-based ROIs to generate the final synaptic and extra-synaptic ROIs used for analysis; FasII fluorescence intensity was measured in these ROIs; FasII and Dlg fluorescence intensity and HRP fluorescence area were also measured at each synapse independent of the ROIs; FasII fluorescence intensity/HRP synaptic area was calculated for the whole synapse, the synaptic region, and the extra-synaptic region; the percent extra-synaptic FasII was calculated based on these values.

### Western blotting

SDS-PAGE was performed using the Novex NuPAGE SDS-PAGE system with 4%-12% Bis-Tris gels run at 125 V for 5 hours to achieve separation of individual FasII bands. Transfer to nitrocellulose membrane (Whatman, Dassel, Germany) was performed using a Trans-Blot-SD Semi-Dry Transfer Cell (Bio-Rad, Hercules, CA). Blocking was performed in 5% BSA for FasII blots or 5% milk for actin blots in 1X PBS with 0.1% Tween 20. Primary antibodies were obtained from the DSHB: mouse anti-FasII (1D4) 1:100 and mouse anti-actin (JLA20) 1:1000. Horseradish peroxidase-conjugated goat anti-mouse secondary antibody (Jackson ImmunoResearch Laboratories, Inc., West Grove, PA) was used at 1:1000 for FasII blots and 1:5000 for actin blots. All antibodies were diluted in blocking buffer. Blots were developed with SuperSignal West Pico Chemiluminescent Substrate (Thermo Scientific, Waltham, MA) and imaged with Amersham Hyperfilm ECL film (GE Healthcare Limited, Buckinghamshire, UK). Band intensity was quantified using ImageJ.

### Data and statistical analyses

Statistical significance was assessed either by Student’s T-Test with direct comparison between an experimental data set and a control data set, or one-way ANOVA with Tukey’s post-hoc test across multiple data sets, as appropriate. Specific *p* value ranges are given in the figure legends, with * *p* < 0.05, ** *p* < 0.01, and *** *p* < 0.001 marked as significant. Some *p* values that may trend toward significance (*p* < 0.1) are also indicated. The values reported in text or plotted on bar graphs are mean ± SEM, with *n* values for experimental trials placed on a bar. Raw values for electrophysiological data–including *n* values for control and exact experimental genotypes–are given in S1 Table.

## Supporting Information

S1 Table Raw electrophysiological data measuring synaptic homeostasis. For each experiment, a homeostatic challenge was utilized (*GluRIII* RNAi-mediated knock down, *GluRIIA*^*SP16*^ mutation, or PhTox). Control genotypes lack a homeostatic challenge and are paired next to genetically matched, challenged experimental genotypes. Values are mean ± SEM. mEPSP (average miniature excitatory postsynaptic potential); EPSP (average excitatory postsynaptic potential); QC (average quantal content); NLS QC (average QC corrected for non-linear summation); I_R_ (average muscle input resistance); RMP (average resting membrane potential); n (number of NMJs recorded for the indicated genotype). Some Student T-Test comparisons to control (usually in row directly above) are provided for QC and NLS QC, highlighting results in the main text and figures. Specific *p* values or *p* value ranges match those in the text, figures, and figure legends, with *p* < 0.05 marked as significant (^#^ *p* = 0.05; * *p* < 0.05; ** *p* < 0.01; *** *p* < 0.001). Statistical tests marked with an accompanying ‘(low)’ symbol indicated that QC or NLS QC is actually significantly depressed from baseline when there is a homeostatic challenge. Various GAL4 drivers were used for *GAL4/UAS* expression experiments. The lines (and tissues) abbreviated in the table are as follows. All GAL4 drivers were used in a heterozygous condition. *C155 –elaV(C155)-Gal4* (pan-neuronal, post-mitotic neurons), *Sca–Scabrous-Gal4* (neuronal), *OK371 –OK371-Gal4* (motor neurons), *BG57 –BG57-Gal4* (also known as *C57-Gal4*; muscles), *MHC–MHC-Gal4* (muscles), *MyoD–MyoD-Gal4* (also known as *nau-Gal4*; muscles).
(PDF)

S1 Fig Morphological analysis of selected genetic manipulations. For all morphological data, values are shown for third instar larvae raised at 25°C **(A-C)** or at 29°C **(D-F)**. **(A, D)** Total bouton numbers at the muscle 6/7 synapse in abdominal segments 2 and 3 (A2, A3) **(B, E)** Muscle area (pixels^2^) for the synapses analyzed in A and D. **(C, F)** Bouton number normalized to muscle area. Challenged genotypes (*GluRIIA* or *GluRIII-KD*) are not significantly different from their genetic controls unless otherwise indicated in the figure. ^#^ p ≤ 0.07, * *p* < 0.05, ** *p* < 0.01, *** *p* < 0.001, ns—not significant (*p* > 0.1) by Student’s T-test compared to control.
(TIF)
