# Supplementary material for: C-terminal Src Kinase Gates Homeostatic Synaptic Plasticity and Regulates Fasciclin II Expression at the Drosophila Neuromuscular Junction
Source: PLoS Genet. 2016 Feb 22;12(2):e1005886. doi: 10.1371/journal.pgen.1005886 (PMC4764653; doi:10.1371/journal.pgen.1005886)
Supplement: S1 Table — For each experiment, a homeostatic challenge was utilized (GluRIII RNAi-mediated knock down, GluRIIASP16 mutation, or PhTox). Control genotypes lack a homeostatic challenge and are paired next to genetically matched, challenged experimental genotypes. Values are mean ± SEM. mEPSP (average miniature excitatory postsynaptic potential); EPSP (average excitatory postsynaptic potential); QC (average quantal content); NLS QC (average QC corrected for non-linear summation); IR (average muscle input resistance); RMP (average resting membrane potential); n (number of NMJs recorded for the indicated genotype). Some Student T-Test comparisons to control (usually in row directly above) are provided for QC and NLS QC, highlighting results in the main text and figures. Specific p values or p value ranges match those in the text, figures, and figure legends, with p < 0.05 marked as significant (# p = 0.05; * p < 0.05; ** p < 0.01; *** p < 0.001). Statistical tests marked with an accompanying ‘(low)’ symbol indicated that QC or NLS QC is actually significantly depressed from baseline when there is a homeostatic challenge. Various GAL4 drivers were used for GAL4/UAS expression experiments. The lines (and tissues) abbreviated in the table are as follows. All GAL4 drivers were used in a heterozygous condition. C155 –elaV(C155)-Gal4 (pan-neuronal, post-mitotic neurons), Sca–Scabrous-Gal4 (neuronal), OK371 –OK371-Gal4 (motor neurons), BG57 –BG57-Gal4 (also known as C57-Gal4; muscles), MHC–MHC-Gal4 (muscles), MyoD–MyoD-Gal4 (also known as nau-Gal4; muscles). (PDF) [file pgen.1005886.s001.pdf]

Genotype	mEPSP (mV)	EPSP (mV)	QC (Stats *)	NLS QC (Stats *)	I_R (MΩ)	RMP (mV)	n	Fig
w^{1118} (Wild Type)	0.86 ± 0.05	32.7 ± 1.4	40.5 ± 2.4	74.4 ± 5.8	6.4 ± 0.4	-63.3 ± 0.9	23	
w; GluRIIA^{SP16}	0.34 ± 0.01	22.3 ± 0.7	**66.9 ± 3.0** ***	**97.0 ± 4.9** **	7.0 ± 0.5	-63.9 ± 0.7	19	1,3,4 6,7
w^{1118}*, PhTx 10 min*	0.41 ± 0.02	29.3 ± 1.1	**73.6 ± 5.8** ***	**125.5 ± 12.2** **	5.0 ± 0.3	-62.9 ± 0.5	11	1
**Muscle and Neurons:** *C155/Y; Sca/+; BG57/+*	0.83 ± 0.06	37.9 ± 1.0	48.5 ± 3.4	97.9 ± 7.5	8.8 ± 0.4	-66.0 ± 0.9	15	4
C155/Y; Sca/+; BG57, GluRIII-RNAi/+	0.48 ± 0.02	34.5 ± 0.8	**73.9 ± 2.7** ***	**139.7 ± 5.4** ***	7.3 ± 0.6	-63.7 ± 0.4	25	1,4,6
C155/Y; Sca/+; BG57/Csk-RNAi	0.92 ± 0.06	34.8 ± 1.3	39.0 ± 2.3	71.7 ± 5.0	5.9 ± 0.3	-66.9 ± 1.3	10	
C155/Y; Sca/+; BG57, GluRIII-RNAi/Csk-RNAi	0.54 ± 0.04	23.4 ±2.4	**43.0 ± 3.4** ^{ns}	**65.8 ± 7.4** ^{ns}	5.8 ± 0.3	-65.4 ± 1.2	13	1
Csk^{c04256}	0.80 ± 0.07	36.4 ± 1.8	47.9 ± 5.4	89.1 ± 12.0	6.0 ± 0.3	-70.5 ± 1.7	11	3
GluRIIA^{SP16}*; Csk*^{c04256}	0.27 ± 0.01	15.7 ± 1.3	**58.0 ± 4.7** ^{ns}	**75.7 ± 7.5** ^{ns}	5.7 ± 0.4	-63.2 ± 0.8	11	1
Csk^{c04256}*, PhTx 10 min*	0.47 ± 0.03	35.3 ± 2.0	**76.5 ± 4.2** ***	**141.0 ± 11.4** **	5.3 ± 0.3	-68.9 ± 1.2	10	1
Csk^{c04256}*/Csk*^{j1D8}	0.76 ± 0.06	29.7 ± 1.2	40.9 ± 2.6	67.1 ± 4.4	5.6 ± 0.3	-66.8 ± 1.0	10	3
GluRIIA^{SP16}*; Csk*$^{c04256/j1D8}$	0.39 ± 0.02	14.1 ± 1.8	**37.2 ± 5.2** ^{ns}	**47.1 ± 7.2** $^{*(low)}$	8.8 ± 0.7	-68.5 ± 1.5	10	1
cac^{S}*/+*	0.74 ± 0.02	29.9 ± 1.4	42.1 ± 3.0	74.0± 7.0	5.0 ± 0.2	-62.8 ± 0.6	25	
cac^{S}*/+; GluRIIA*^{SP16}	0.32 ± 0.01	15.9 ± 0.09	**50.4 ± 3.3** *	66.3 ± 5.1	5.8 ± 0.2	-67.8 ± 0.8	31	1
Csk^{j1D8}*/+*	0.85 ± 0.08	32.1 ± 1.5	41.1 ± 4.0	75.6 ± 8.7	6.5 ± 0.7	-63.7 ± 0.8	10	
GluRIIA^{SP16}*; Csk*^{j1D8}*/+*	0.32 ± 0.02	18.9 ± 1.2	**61.7 ± 5.3** **	84.9 ± 8.7	7.7 ± 0.7	-62.8 ± 0.7	11	1
cac^{S}*/+;; Csk*^{j1D8}*/+*	0.71 ± 0.02	32.2 ± 0.1	45.8 ± 2.0	82.4 ± 4.8	4.6 ± 0.3	-63.5 ± 0.6	10	
cac^{S}*/+; GluRIIA*^{SP16}*; Csk*^{j1D8}*/+*	0.30 ± 0.02	9.8 ± 1.4	**33.6 ± 5.1** $^{*(low)}$	**40.0 ± 6.6** $^{***(low)}$	6.8 ± 0.6	-62.9 ± 0.8	11	1
**Src64B Muscle:** *UAS-Src64B*^{UY1332}*/BG57* 25°C	0.80 ± 0.05	35.3 ± 2.3	45.0 ± 2.7	96.7 ± 15.0	6.4 ± 0.4	-65.9 ± 3.9	9	
GluRIIA^{SP16}*; UAS-Src64B*^{UY1332}*/BG57* 25°C	0.33 ± 0.01	17.2 ± 1.8	**52.0 ± 4.8** ^{ns}	**63.1 ± 8.2** ^{ns}	7.3 ± 0.4	-66.2 ± 0.9	8	4
**Src64B Neur.:** *C155; UAS-Src64B*^{UY1332} 25°C	1.08 ± 0.06	35.4 ± 1.5	33.4 ± 2.3	64.2 ± 6.2	7.9 ± 0.8	-65.7 ± 1.6	10	
C155; GluRIIA^{SP16}*; UAS-Src64B*^{UY1332} 25°C	0.43 ± 0.6	21.7 ± 2.1	**60.6 ± 11.5** *	**89.1 ± 19.2** $^{p=0.2}$	7.5 ± 1.1	-65.7 ± 1.2	8	4
**Src42A Muscle:** *UAS-Src42A*^{WT}*; BG57/+* 18°C	0.69 ± 0.03	31.1 ± 1.0	45.4 ± 2.2	80.3 ± 5.6	5.2 ± 0.4	-63.0 ± 0.8	9	
UAS-Src42A^{WT}*; GluRIIA*^{SP16}*; BG57/+* 18°C	0.30 ± 0.02	15.8 ± 1.2	**54.2 ± 4.4** ^{ns}	**70.4 ± 7.2** ^{ns}	5.1 ± 0.4	-61.9 ± 0.5	9	4
**Src42A Muscle:** *UAS-Src42A*^{YF}*; MHC/+ 18°C*	0.77 ± 0.07	31.5 ± 3.5	42.1 ± 5.2	75.9 ± 12.0	7.9 ± 0.7	-65.9 ± 1.6	7	
UAS-Src42A^{YF}*; GluRIIA*^{SP16}*; MHC/+* 18°C	0.33 ± 0.02	17.0 ± 2.0	**56.3 ± 8.1** ^{ns}	**77.6 ± 13.5** ^{ns}	8.0 ± 0.7	-62.9 ± 0.6	10	4
**Src 42A Neur:** *OK371/+; UAS-Src42A*^{YF}*/+* 18°C	0.89 ± 0.07	32.9 ± 1.2	38.7 ± 3.2	69.5 ± 6.4	5.8 ± 0.6	-65.0 ± 0.9	10	
OK371, GluRIIA^{SP16} */+, GluRIIA*^{SP16}*; UAS-Src42A*^{YF}*/+* 18°C	0.35 ± 0.02	27.3 ± 1.9	**81.0 ± 8.2** *	**134.6 ± 17.1** **	9.3 ± 0.8	-62.0 ± 0.6	7	4
Src42A^{k10108}*/+; Csk*^{c04256}	0.75 ± 0.05	32.7 ± 1.7	45.4 ± 4.2	83.7 ± 7.7	5.1 ± 0.3	-62.9 ± 0.6	9	
Src42A^{k10108}*, GluRIIA*^{SP16}*/+, GluRIIA*^{SP16}*; Csk*^{c04256}	0.37 ± 0.02	19.5 ± 5.0	**54.2 ± 8.7** ^{ns}	84.0 ± 11.5	5.9 ± 0.5	-66.7 ± 0.9	12	4

Genotype	mEPSP (mV)	EPSP (mV)	QC (Stats *)	NLS QC (Stats *)	I_R (MΩ)	RMP (mV)	n	Fig
Src64B^{KO}, *Csk*^{c04256}/+, *Csk*^{j1D8}	0.63 ± 0.05	29.7 ± 1.7	48.2 ± 4.2	82.8 ± 8.5	4.1 ± 0.1	-63.1 ± 0.7	7	
GluRIIA^{SP16}; *Src64B*^{KO}, *Csk*^{c04256}/+, *Csk*^{j1D8}	0.32 ± 0.02	19.8 ± 1.1	**63.8 ± 3.9** *	86.9 ± 6.4	6.9 ± 0.4	-66.4 ± 1.1	14	4
Src42A^{k10108}/+	0.89 ± 0.07	38.2 ± 1.1	44.9 ± 3.2	89.8 ± 7.1	6.3 ± 0.6	-67.0 ± 1.2	10	
Src42A^{k10108}, *GluRIIA*^{SP16}/+, *GluRIIA*^{SP16}	0.41 ± 0.03	25.5 ± 2.8	**64.3 ± 7.0** **	101.5 ± 12.9	6.8 ± 0.9	-67.9 ± 1.7	14	4
Src64B^{KO}/+	0.90 ± 0.06	36.4 ± 1.6	41.3 ± 2.1	79.8 ± 5.2	5.8 ± 0.5	-66.0 ± 1.4	9	
GluRIIA^{SP16}; *Src64B*^{KO}/+	0.42 ± 0.04	22.2 ± 2.5	**58.6 ± 8.6** $^{p=0.08}$	89.2 ± 14.7	6.7 ± 0.5	-64.3 ± 0.9	10	4
**Musc & Neur:** *UAS-Csk-YFP/C155; Sca/+; BG57/+*	1.04 ± 0.06	26.1 ± 0.2	25.2 ± 0.6	39.7 ± 1.0	6.9 ± 0.1	-66.2 ± 0.2	11	5
UAS-Csk-YFP/C155; Sca/+; BG57, GluRIII-RNAi/+	0.52 ± 0.02	28.5 ± 3.5	**54.7 ± 6.6** ***	**91.8 ± 14.5** ***	5.7 ± 0.3	-65.8 ± 1.1	9	5
UAS-Csk-YFP; Csk^{c04256}/*Csk*^{j1D8}	0.86 ± 0.03	36.3 ± 1.5	42.8 ± 2.2	83.3 ± 6.6	6.6 ± 0.3	-66.5 ± 1.1	11	
UAS-Csk-YFP; GluRIIA^{SP16}; *Csk*$^{c04256/j1D8}$	0.38 ± 0.01	13.5 ± 1.7	**35.1 ± 4.4** ^{ns}	**44.2 ± 6.3** $^{***(low)}$	7.8 ± 0.5	-63.4 ± 0.7	8	5
**Mus Rescue:** *UAS-Csk-YFP; MyoD/+; Csk*$^{c04256/j1D8}$	0.96 ± 0.07	35.0 ± 0.08	38.3 ± 3.6	72.6 ± 8.1	5.3 ± 0.4	-65.2 ± 0.4	9	
UAS-Csk-YFP; MyoD het, GluRIIA^{SP16}; *Csk*$^{c04256/j1D8}$	0.43 ± 0.02	28.1 ± 1.7	**67.1 ± 7.2** **	**110.9 ± 16.0** *	6.1 ± 0.4	-65.2 ± 1.4	8	5
**Neur. Rescue:** *UAS-Csk-YFP*; *OK371/+*; *Csk*$^{c04256/j1D8}$	1.05 ± 0.08	40.8 ± 1.1	40.5 ± 3.1	80.7 ± 7.6	4.7 ± 0.3	-72.6 ± 1.2	8	
UAS-Csk-YFP; OK371, GluRIIA^{SP16}/+, *GluRIIA*^{SP16}; *Csk*$^{c04256/j1D8}$	0.45 ± 0.4	26.6 ± 1.0	**62.4 ± 6.0** **	99.1 ± 11.3	6.3 ± 0.6	-63.5 ± 1.0	8	5
**Muscle RNAi:** *BG57/Csk-RNAi*	0.71 ± 0.06	33.3 ± 1.8	48.3 ± 2.9	85.1 ± 6.6	5.9 ± 0.6	-68.9 ± 1.3	8	
GluRIIA^{SP16}; *BG57/Csk-RNAi*	0.36 ± 0.02	21.7 ± 1.3	**62.9 ± 5.5** *	**90.8 ± 9.6** ^{ns}	6.1 ± 0.2	-64.8 ± 1.6	11	5
**Neuronal RNAi:** *C155*/Y; *Csk-RNAi/+*	0.81 ± 0.03	32.8 ± 1.5	42.1 ± 3.1	79.9 ± 8.2	4.6 ± 0.2	-62.0 ± 0.4	7	
C155/Y: *GluRIIA*^{SP16}; *Csk-RNAi/+*	0.37 ± 0.03	26.6 ± 1.0	**77.3 ± 8.4** ***	**124.6 ± 15.3 ***	5.9 ± 0.7	-62.3 ± 0.5	9	5
**Musc & Neur:** *C155/FasII*^{EP1462}; *Sca/+; BG57/+*	0.57 ± 0.1	34.3 ± 1.1	63.1 ± 5.0	118.4 ± 10.1	4.9 ± 0.2	-63.9 ± 0.8	8	
C155/FasII^{EP1462}; *Sca/+; BG57, GluRIII-RNAi/+*	0.36 ± 0.02	27.5 ± 0.7	**78.5 ± 4.8** *	126.0 ± 7.8	5.3 ± 0.5	-63.1 ± 0.6	8	8
**Muscle FasII O/E:** *FasII*^{EP1462}; *BG57/+*	0.48 ± 0.02	33.2 ± 1.2	69.9 ± 3.4	124.3 ± 6.5	5.4 ± 0.6	-66.4 ± 1.7	9	
FasII^{EP1462}; *GluRIIA*^{SP16}; *BG57/+*	0.22 ± 0.01	19.9 ± 1.3	**90.5 ± 6.0** *	125.7 ± 11.5	4.4 ± 0.3	-63.7 ± 1.2	8	8
**Neuron FasII O/E:** *C155/FasII*^{EP1462}	1.01 ± 0.27	33.8 ± 1.4	41.7 ± 4.6	77.8 ± 9.0	5.5 ± 0.5	-63.1 ± 1.2	8	
C155/FasII^{EP1462}; *GluRIIA*^{SP16}	0.32 ± 0.02	19.5 ± 1.6	**64.3 ± 8.6** *	91.2 ± 15.4	6.0 ± 0.5	-63.9 ± 1.0	9	8
C155/Y; UAS-FasIIA-PEST+/Sca; BG57/+	0.74 ± 0.06	36.0 ± 3.1	50.1 ± 4.2	98.3 ± 11.7	8.1 ± 0.9	-67.1 ±1.1	8	
C155/Y; UAS-FasIIA-PEST+/Sca; BG57, GluRIII-RNAi/+	0.38 ± 0.03	29.6 ± 2.2	**80.1 ± 8.4** **	131.7 ± 16.3	11.3 ± 0.9	-66.6 ± 3.3	8	8
C155/Y; UAS-FasIIA-PEST-/Sca; BG57/+	0.72 ± 0.3	33.7 ± 2.3	46.8 ± 3.1	87.6 ± 8.8	7.1 ± 0.3	-65.5 ± 1.2	8	
C155/Y; UAS-FasIIA-PEST-/Sca; BG57, GluRIII-RNAi/+	0.56 ± 0.02	35.3 ± 1.2	**63.6 ± 2.8** **	**121.4 ± 7.0** *	8.6 ± 0.8	-64.8 ± 0.9	8	8

C155/Y; UAS-FasIIC/Sca; BG57/+	0.63 ± 0.08	24.2 ± 2.2	40.0 ± 2.9	60.5 ± 4.8	11.1 ± 1.4	-62.3 ± 0.9	8	
C155/Y; UAS-FasIIC/Sca; BG57, GluRIII-RNAi/+	0.36 ± 0.04	23.6 ± 1.8	**70.1 ± 2.6****	**107.9 ± 13.5****$^{}$	8.7 ± 0.9	-60.1 ± 0.5	9	8
FasII^{EP1462}/UAS-Csk-YFP;; BG57/+	0.50 ± 0.03	30.4 ± 1.6	61.0 ± 2.4	100.8 ± 4.8	6.1 ± 0.4	-67.3 ± 1.9	8	
FasII^{EP1462}/UAS-Csk-YFP; GluRIIA^{SP16}; BG57 /+	0.29 ± 0.03	16.7 ± 1.0	61.5 ± 6.3	79.6 ± 8.8	7.0 ± 0.8	-66.4 ± 2.5	8	8
FasII^{e76}/Y	1.17 ± 0.11	31.8 ± 1.0	28.4 ± 1.9	51.3 ± 3.7	7.8 ± 0.5	-61.7 ± 0.5	9	
FasII^{e76}/Y; BG57, GluRIII-RNAi/+	0.60 ± 0.03	31.5 ± 1.3	**53.6 ± 2.9*****	**97.7 ± 8.3****	6.5 ± 0.3	-62.2 ± 0.4	12	8
FasII^{e76}/FasII^{G293}	0.75 ± 0.06	33.0 ± 1.1	45.8 ± 3.2	84.6 ± 6.0	6.4 ± 0.5	-62.4 ± 0.8	9	
FasII^{e76}/FasII^{G293}; BG57, GluRIII-RNAi/+	0.44 ± 0.02	29.2 ± 1.7	**66.1 ± 3.8****	**112.8 ± 9.7***	7.1 ± 0.4	-62.6 ± 0.5	9	8
C155/Y; FasII-RNAi^{JF02918}/Sca; BG57/+	0.70 ± 0.04	30.7 ±1.5	44.9 ± 2.1	77.6 ± 5.1	6.3 ± 0.4	-64.0 ±0.8	8	
C155/Y; FasII-RNAi^{JF02918}/Sca; BG57, GluRIII-RNAi/+	0.42 ± 0.2	25.9 ± 1.4	**62.9 ± 3.8***	**99.8 ± 7.8***	6.0 ± 3.8	-62.0 ± 0.5	11	8
C155/Y; FasII-RNAi^{v36351}/Sca; BG57/+	0.67 ± 0.04	25.6 ± 1.9	39.7 ± 0.1	63.1 ± 7.0	5.0 ± 0.3	-62.4 ± 0.5	9	
C155/Y; FasII-RNAi^{v36351}/Sca; BG57, GluRIII-RNAi/+	0.33 ± 0.03	27.0 ± 1.0	**85.3 ± 6.2*****	**135.8 ± 10.4*****	4.4 ± 0.3	-62.5 ± 0.8	7	8
FasII^{e76}; Csk$^{c04256/j1D8}$	0.94 ± 0.10	37.8 ± 1.5	42.6 ± 3.4	85.0 ± 5.4	6.9 ± 0.5	-65.6 ± 1.1	8	
FasII^{e76}; GluRIIA^{SP16}; Csk$^{c04256/j1D8}$	0.48 ± 0.01	29.1 ± 1.9	**60.1 ± 3.5****	99.5 ± 9.3	7.9 ± 0.7	-66.2 ± 1.2	8	9

S1 Table: Raw electrophysiological data measuring synaptic homeostasis. For each experiment, a homeostatic challenge was utilized (*GluRIII* RNAi-mediated knock down, *GluRIIA^{SP16}* mutation, or PhTox). Control genotypes lack a homeostatic challenge and are paired next to genetically matched, challenged experimental genotypes. Values are mean ± SEM. mEPSP (average miniature excitatory postsynaptic potential); EPSP (average excitatory postsynaptic potential); QC (average quantal content); NLS QC (average QC corrected for non-linear summation); I_R (average muscle input resistance); RMP (average resting membrane potential); n (number of NMJs recorded for the indicated genotype). Some Student T-Test comparisons to control (usually in row directly above) are provided for QC and NLS QC, highlighting results in the main text and figures. Specific *p* values or *p* value ranges match those in the text, figures, and figure legends, with $p < 0.05$ marked as significant ($^{\#}$ $p = 0.05$; * $p < 0.05$; ** $p < 0.01$; *** $p < 0.001$). Statistical tests marked with an accompanying '(low)' symbol indicated that QC or NLS QC is actually significantly depressed from baseline when there is a homeostatic challenge.

Various GAL4 drivers were used for *GAL4/UAS* expression experiments. The lines (and tissues) abbreviated in the table are as follows. All GAL4 drivers were used in a heterozygous condition.

C155 – elaV(C155)-Gal4 (pan-neuronal, post-mitotic neurons)
Sca – Scabrous-Gal4 (neuronal)
OK371 – OK371-Gal4 (motor neurons)
BG57 – BG57-Gal4 (also known as *C57-Gal4*; muscles)
MHC – MHC-Gal4 (muscles)
MyoD – MyoD-Gal4 (also known as *nau-Gal4*; muscles)
