# Supplementary figures and images for: C-terminal Src Kinase Gates Homeostatic Synaptic Plasticity and Regulates Fasciclin II Expression at the Drosophila Neuromuscular Junction
Source: PLoS Genet. 2016 Feb 22;12(2):e1005886. doi: 10.1371/journal.pgen.1005886 (PMC4764653; doi:10.1371/journal.pgen.1005886)

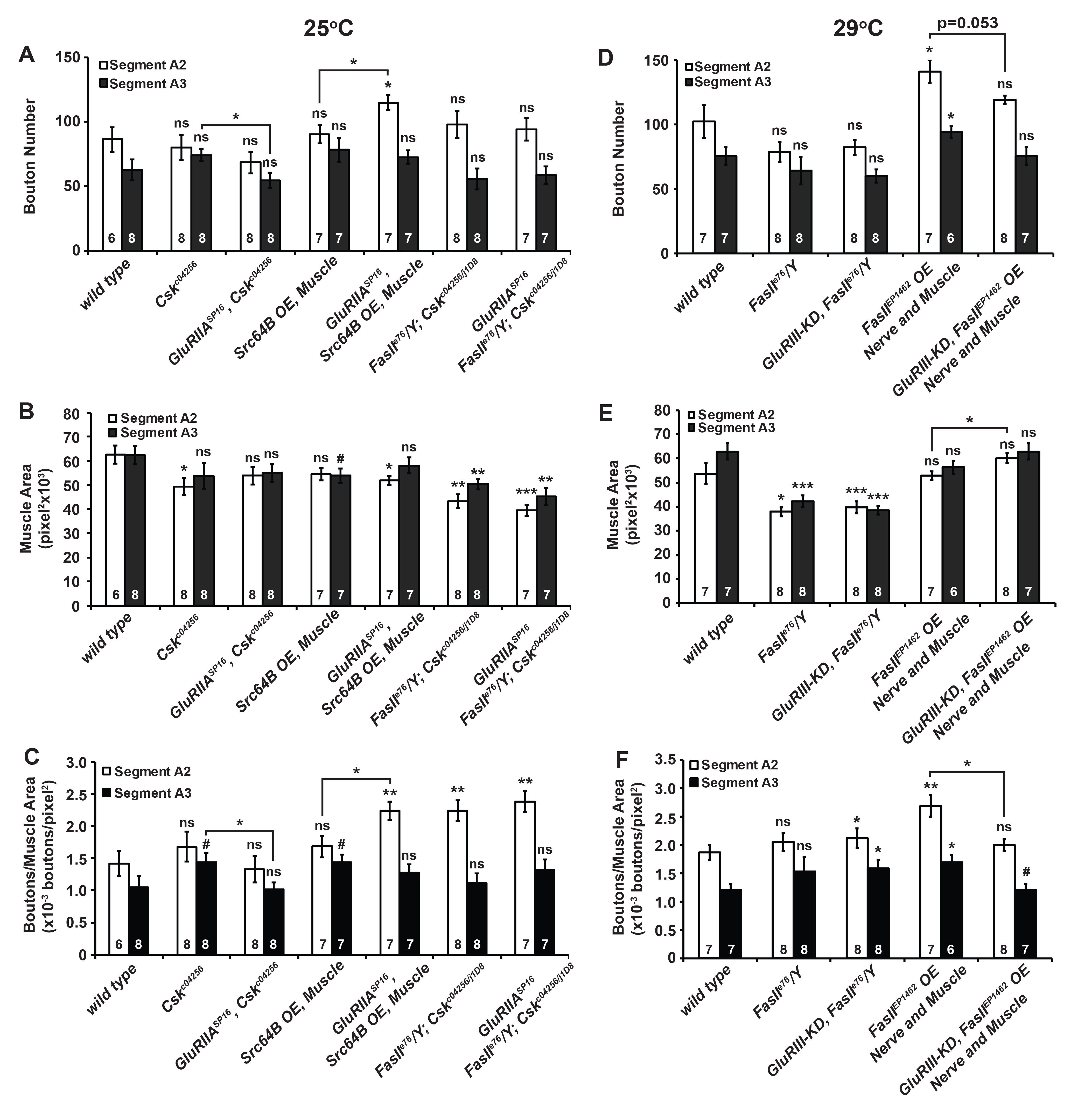

Supplement: S1 Fig — For all morphological data, values are shown for third instar larvae raised at 25°C (A-C) or at 29°C (D-F). (A, D) Total bouton numbers at the muscle 6/7 synapse in abdominal segments 2 and 3 (A2, A3) (B, E) Muscle area (pixels2) for the synapses analyzed in A and D. (C, F) Bouton number normalized to muscle area. Challenged genotypes (GluRIIA or GluRIII-KD) are not significantly different from their genetic controls unless otherwise indicated in the figure. # p ≤ 0.07, * p < 0.05, ** p < 0.01, *** p < 0.001, ns—not significant (p > 0.1) by Student’s T-test compared to control. (TIF) [file pgen.1005886.s002.tif]
